# Umbrella review of psychosocial and ward-based interventions to reduce self-harm and suicide risks in in-patient mental health settings

**DOI:** 10.1192/bjo.2025.10811

**Published:** 2025-09-08

**Authors:** Leah Quinlivan, Jodie Westhead, Jane Graney, Fanyi Su, Sarah Steeg, Emma Nielsen, Eloise Curtis, Ellie Wildbore, Faraz Mughal, Rachel Elliott, Roger T. Webb, Nav Kapur

**Affiliations:** Division of Psychology and Mental Health, University of Manchester, UK; Manchester Academic Health Science Centre, University of Manchester, UK; National Institute for Health and Care Research (NIHR GM PSRC) Greater Manchester Patient Safety Research Collaboration, University of Manchester, UK; Manchester Centre for Health Economics, University of Manchester, UK; School of Medicine, Keele University, UK; College of Medicine and Health, University of Birmingham, UK; Mersey Care NHS Foundation Trust, Manchester, UK

**Keywords:** Self-harm, psychosocial interventions, suicide, therapeutic interventions, umbrella review

## Abstract

**Background:**

Understanding what psychosocial interventions can reduce self-harm and suicide within in-patient mental health settings can be challenging, due to clinical demands and the large volume of published reviews.

**Aims:**

To summarise evidence from systematic reviews on psychosocial and ward-level interventions (excluding environmental modifications) for self-harm and suicide that may enhance patient safety in in-patient mental health settings.

**Method:**

We systematically searched Medline, Embase, CINAHL, PsycINFO and CDSR (2013–2023) for systematic reviews on self-harm and suicide prevention interventions that included in-patient data. Review quality was assessed using AMSTAR-2, primary study overlap via an evidence matrix, and evidence strength evaluated (GRADE algorithm). Findings were narratively synthesised, with input from experts-by-experience throughout (PROSPERO ID: CRD42023442639).

**Results:**

Thirteen systematic reviews (seven meta-analyses, six narrative), comprising over 160 000 participants, were identified. Based on quantitative reviews, cognitive–behavioural therapy reduces repeat self-harm by follow-up, and dialectical behaviour therapy decreases the frequency of self-harm. Narrative review evidence suggested that post-discharge follow-up, as well as system and ward-based interventions (e.g. staff training) may reduce suicide and/or self-harm. However, review quality varied, patient involvement was lacking and methodological quality of trials informing reviews was predominately low. Overlap was slight (covered area 12.4%).

**Conclusions:**

The effectiveness of interventions to prevent self-harm and suicide in in-patient settings remains uncertain due to variable quality reviews, evidence gaps, poor methodological quality of primary studies and a lack of pragmatic trials and co-production. There is an urgent need for better, co-designed research within in-patient mental health settings.

Suicide and self-harm are global public health priorities, with an estimated 700 000 lives lost to suicide and at least 14 million episodes of self-harm occurring annually.^1,2^ In the UK, over 69 000 individuals died by suicide between 2011 and 2021.^3^ Approximately one-third of these individuals were mental health patients, and 6% of these deaths occurred during in-patient care for patients aged 16 years and older.^3,4^ Patients discharged from hospital have an elevated suicide risk.^4,5^ Around 12% of patient deaths occur within three months post-discharge, and risk of suicide is particularly high within the first two weeks.^3–5^ Self-harm, defined as self-injury or self-poisoning irrespective of suicidal intent,^6^ is strongly associated with suicide and is therefore a major patient safety concern in mental health services.^7^ Approximately 20% of individuals presenting to hospital following an episode of self-harm are admitted to in-patient mental health services worldwide.^8^ In the UK, 76% of mental health in-patients who died by suicide had a history of self-harm.^4^ Timely, evidence-based and compassionately delivered interventions are essential to prevent self-harm repetition and suicide.^9^

Mental health in-patient suicide rates have reduced since 2009, but progress in England has stagnated since 2016.^4^ Structural changes, including enhanced ward safety and ligature point removal, may have contributed to improved patient safety^4^ and evidence beyond physical safety measures may further reduce suicide rates in this setting. Psychosocial interventions that involve structured, non-pharmacological treatments with a psychological or social focus, can mitigate self-harm and suicide risk,^10,11^ but access to therapeutic therapies remains limited.^12,13^ Evaluating the suitability and effectiveness of self-harm and suicide prevention interventions for in-patient care is further complicated by clinical demands, information overload and limited evidence translation into practice.^14,15^ Umbrella reviews provide an opportunity to synthesise broad systematic review evidence to guide clinical practice.^16^

Our objective was to summarise the evidence from systematic reviews on psychosocial and ward-level interventions for preventing self-harm and suicide that may enhance patient safety for adults (aged 16 years and older) in in-patient mental health settings. We sought to evaluate the quality and relevance of this systematic review evidence for mental health in-patient settings to inform practice.

## Method

This study is reported according to the Preferred Reported Items for Overviews of Reviews (PRIOR) guideline,^17^ and was registered on the International Prospective Register of Systematic Reviews (PROSPERO) (registration number: CRD42023442639; 5.06.23). Minor changes included utilising the Adapted Algorithm for GRADE,^18^ in place of Guyatt et al^19^ to evaluate the methodological quality and certainty of the evidence.

### Inclusion and exclusion criteria

Reviews were eligible for inclusion if they were: (a) peer reviewed systematic reviews; (b) they included at least one primary study that evaluated psychosocial interventions based in mental health in-patient settings (wards or post-discharge services); (c) reported data for adults aged 18 or over, or composite results for adults and adolescents. Our included outcomes were consistent with clinical guidelines for self-harm,^6^ and included any self-harm, or self-injury irrespective of suicidal intent (See Table 1). Suicidal ideation, while important, was excluded due to our focus on behaviour.^10,20^ We did not have restrictions on study designs, comparators, or psychiatric diagnoses, but we prioritised reviews which focused on interventions which went beyond the purely environmental such as the removal of ligature points. Exclusion criteria included: (a) suicidal ideation (as the composite or main outcome); (b) studies that only reported data for children and adolescents; (c) studies based in prisons and other custodial criminal justice institutions, as well as educational, community or voluntary settings; (d) theoretical and opinion-based reviews, letters, commentaries, non-systematic reviews and reviews of qualitative research; and (e) review articles that were not translated to English.

**Table 1:** PICO^a^ (population, intervention, comparison and outcomes) criteria

Population	Patients who have received self-harm or suicide prevention interventions in mental health in-patient settings (wards or post-in-patient discharge services) (adults or studies that reported composite results for adults and adolescents).
Intervention^a^	**Psychosocial interventions**: Interventions in systematic reviews may have included, but were not limited to: cognitive behavioural therapy-based psychotherapy; dialectical behaviour therapy; mentalisation-based therapy; case management; group-based psychotherapy; brief contact interventions; enhanced assessment approaches; treatment adherence/ compliance enhancement approaches; family interventions; remote contact interventions; and other multimodal interventions. **Ward-level interventions**: ward-based changes, integrated care services, or age-specific services (e.g. older adults). **Training**: Any self-harm or suicide prevention training for mental health in-patient settings, reporting self-harm and/or suicide as outcomes.
Comparison	**Psychosocial or psychological therapy comparisons**: Standard care (e.g. treatment as usual), any other comparator or none (e.g. pre–post designs). **Models of care comparisons**: usual models of care, no changes; or any other comparator; Staff training: No training, usual practice.
Outcome	Self-harm, defined as any intentional act of self-poisoning or self-injury, irrespective of suicidal intent or motivation. This definition also includes attempted suicide and non-suicidal self-injury, because suicidal intent varies within and between episodes. Irrespective of suicidal intent, self-harm is a major risk factor for subsequent adverse events, including suicide. Consistent with the Office of National Statistics for England and Wales, suicide is defined as any death caused by intentional self-harm with or without suicidal intent, or of undetermined intent (ICD-10 codes: X60-X84, Y10-Y34, Y87.2).

a. PICO categories were based on clinical guidelines for self-harm.^6^

### Search strategy and selection criteria

Search strategy details (e.g. terms, reasons for exclusion and additional references) are in the Supplementary materials available at https://doi.org/10.1192/bjo.2025.10811. We searched Embase, PsycInfo, MEDLINE, CINAHL and the Cochrane Database of Systematic Reviews (CDSR) from January 2013 to December 2023, using broad search term strings to capture reviews in this area. Search terms were developed with a specialist librarian and content experts, and adapted from the clinical guidelines and Cochrane reviews of interventions for self-harm.^6,21^ We used forward and backward citation chaining to supplement database searches. Two reviewers (L.Q., J.W.) independently reviewed all the titles, abstracts and full texts of potentially eligible studies, F.M. checked a random 10%, and J.G. cross-checked 100% of the data extracted. Disagreements were resolved via consensus and discussion with senior authors (R.T.W., N.K., R.E.), the wider team (F.M., J.W., F.S.) and our PPIE group (MS4MH-R) members.

### Data extraction

Data extraction was performed in duplicate (L.Q., J.W.) using a standardised form, verified (J.G., F.M.) and reviewed by a multidisciplinary team. We extracted study characteristics, including author details, population, author-defined interventions, comparison, and outcome (PICO), methods, evaluation of bias, heterogeneity, GRADE assessment and results.

### Quality assessment

Two researchers (L.Q., J.W.) independently evaluated the methodological quality of systematic reviews using the Assessment of Multiple Systematic Reviews Tool (AMSTAR-2).^22^ Each of the 16 items is evaluated as either positive (yes), negative (no), or partial positive. Based on seven ‘critical’ and nine ‘non-critical’ domains, we classified reviews into ‘high’, ‘moderate’, ‘low’ and ‘critically low’ quality. Using stringent criteria, reviews with a partial yes, or that did not report data for the AMSTAR 2^22^ item were classified as ‘No’ for domain classifications. In accordance with AMSTAR-2 guidance, reviews that listed justifications for excluded references in a summary format, rather than as a list of individual citations, were marked as negative. As part of evidence evaluation, we tabulated and synthesised data for the adapted GRADE algorithm^18^ and other key methodological data. For the adapted GRADE algorithm, quantitative reviews received downgrades based on the assessment of methodological quality, via heterogeneity, number of participants, risk of bias and items from AMSTAR.^18,22^ Reviews were rated as providing a high level of evidence if they received zero downgrades, moderate if one or two downgrades, low if three or four downgrades and very low if the review received five or six downgrades.^18^ We also report any use of GRADE evaluations^19^ in reviews. We based our conclusions on the combined evidence from quantitative and narrative reviews.

### Overlap analysis

Overlap analysis was conducted to examine the degree to which the same primary studies were included in multiple systematic reviews. We estimated the degree of the primary studies, pairwise, overlap in the reviews (fraction of evidence synthesised in two or more reviews) via the covered area (formula: *N/rc*) and corrected covered area (CCA; formula: (*N* – *r*)/((*r × c*) – *r*)) using the open-access Graphical Representation of Overlap for OVErviews open-access tool.^23,24^ *N* is the total number of publications, *r* is the total number of rows (unique primary studies) and cca is the total number of columns (the number of included reviews). The degrees of overlap observed were categorised as slight (CCA 0–5%), moderate (CCA 6–10%), high (CCA 11–15%) and very high overlap (CCA > 15%).^23,24^ One study was removed from overlap analyses due to insufficient information for the extraction of primary studies’ references, which may result in an underestimation of the true degree of overlap.^25^

### Synthesis methods

The purpose of this umbrella review was to descriptively synthesise the systematic review evidence for interventions that may be helpful in preventing self-harm and/or suicide for in-patient mental health settings. Given the high degree of heterogeneity across the set of reviews, we used a systematic approach to narratively describe and synthesise the data in tables and groups.^26^ We grouped systematic review results into those with quantitative (meta-analysis) and narrative synthesis analysis. We summarised the findings from the two groups separately and reported the results and outcomes as described by the review. We reported detailed results for interventions with evidence of efficacy, but also reported contradictory findings and summarised those without. For quantitative reviews, we reported important parameters for significant results including pooled odds ratios, mean differences, 95% CI and means and s.d.s where available. We reported the *I*^2^ statistic as a measure of between-study variation and heterogeneity.^27^ The outcomes were classified into self-harm, attempted suicide and suicide, based on the information available in the publication. Interventions are reported as defined by the systematic reviews (Table 2). We provide detailed information on reviews, including interventions, control groups, study designs and outcomes in Table 4, and additional detailed results are in the Supplementary materials 2, Tables 1 and 3.

## Results

**Fig. 1:**
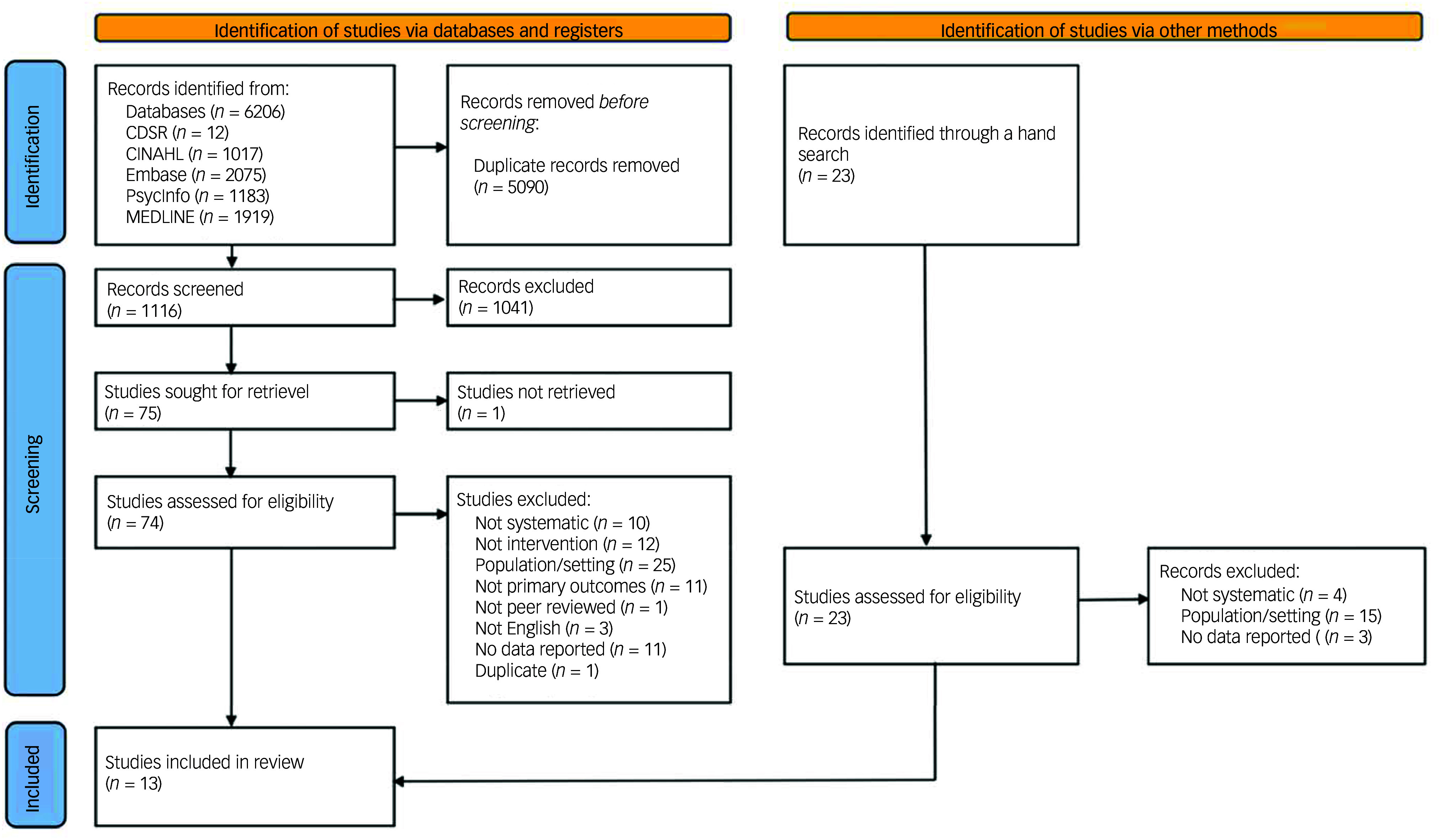
Flow diagram of included studies. CDSR, Cochrane Database of Systematic Reviews.

Fig. 1 summarises the results of the search, which yielded 1116 studies, of which, 1041 were excluded at the title and abstract screening stage. We screened 74 for full text eligibility and identified 23 additional studies through manual searches. In total, after full text screening and stratification, 31 reviews included relevant data for healthcare settings, and 13 met our inclusion criteria for mental health in-patient settings (see Fig. 1 for flow chart).

### Evaluation of bias in reviews

Five studies used the Cochrane Risk of Bias Tool,^21,28–31^ four used another method of evaluating bias (e.g. the Joanna Briggs Institute appraisal tool),^25,32–35^ and three reviews did not report information^10,36,37^ (see Table 2). The predominant concerns for bias included participant attrition and incomplete outcome data. Bias due to the absence of blinding was common but is challenging given the nature of psychosocial interventions.

### Assessment of the reviews’ methodological quality and certainty of the evidence

AMSTAR-2 assessments for the included reviews are presented in Table 2. The methodological quality varied widely across reviews: two reviews were evaluated as providing a ‘high quality’^21,28^ summary of the results and available data, with the remainder rated as ‘critically low’ quality due to more than one critical flaw (e.g. lack of pre-registration and reference list with justifications for each excluded study) (Table 2). According to the adapted GRADE algorithm,^18^ two reviews^21,28^ provided a moderate level of evidence. The risk of bias for included trials in all the reviews resulted in ‘downgrades’ for quality, which reduced the certainty of evidence. For example, Witt et al^21^ conducted a high-quality systematic review, but the primary trials were reported as ‘low quality’ for cognitive-behaviour therapy, moderate certainty for emotion-regulation therapy and ‘very low quality’ for dialectical-behaviour therapy versus treatment as usual. Additional information that contributed to the methodological evaluation of reviews is presented in Table 3.

**Table 2:** AMSTAR-2^22^ ratings across the 13 systematic reviews

Citation	1. PICO	2. Protocol^a^	3. Study design	4. Search strategy^a^	5. Duplicate selection	6. Duplicate extraction	7. Exclude studies listed^a^	8. Included studies	9. Risk of bias (RoB)^a^	10. Funding sources	11. Analysis^a^	12. Impact of Ro	13. RoB in discussion^a^	14. Heterogeneity	15. Publication bias^a^	Conflict of interest	Synthesis type	AMSTAR ranking
Witt^21^	Yes	Yes	Yes	Yes	Yes	Yes	Yes	Yes	Yes	Yes	Yes	Yes	Yes	Yes	Yes	Yes	Meta-analysis	High
Hawton^28^	Yes	Yes	Yes	Yes	Yes	Yes	Yes	Yes	Yes	Yes	Yes	Yes	Yes	Yes	Yes	Yes	Meta-analysis	High
Hou^29^	Yes	No	Yes	Partial	Yes	Yes	No	Yes	Yes	No	Yes	Yes	Yes	Yes	Yes	Yes	Meta-analysis	Critically low
Yiu^31^	Yes	Yes	Yes	Yes	Yes	Yes	No	Yes	Yes	No	Yes	Yes	Yes	Yes	Partial	Yes	Meta-analysis	Critically low
Fox^25^	Yes	No	Yes	Partial	Yes	Yes	No	Partial	Yes	No	Yes	yes	Yes	Partial	Yes	No	Meta-analysis	Critically low
Rozek^33^	Yes	No	Yes	Yes	Yes	Yes	No	Partial	yes	No	NA	NA	No	No	NA	Yes	Narrative	Critically low
Wand^35^	Yes	No	Yes	Partial	Yes	Yes	No	Yes	Yes	No	NA	NA	Yes	Partial	NA	Yes	Narrative	Critically low
Sobanski^30^	Yes	No	Yes	Partial	Yes	No	No	Yes	Yes	No	Yes	No	Yes	Yes	Yes	Yes	Meta-analysis	Critically low
DeCou^36^	Yes	No	Yes	Partial	No	No	No	Yes	No	No	Yes	No	No	Partial	No	Yes	Meta-analysis	Critically low
Donker^34^	Yes	No	Yes	Partial	Yes	Yes	No	Yes	No	No	NA	NA	No	Partial	NA	Yes	Narrative	Critically low
Luxton^37^	Yes	No	No	Partial	No	No	No	Partial	No	No	NA	NA	No	No	NA	No	Narrative	Critically low
Mann^10^	yes	No	Yes	Partial	No	No	No	Yes	Yes	No	NA	NA	No	Yes	NA	Yes	Narrative	Critically low
Nawaz^32^	Yes	Yes	No	Yes	Yes	No	No	Partial	No	No	NA	NA	Yes	No	NA	Yes	Narrative	Critically low

NA, not applicable; PICO, patient, intervention, comparison, outcome.

a. Critical flaws AMSTAR ratings:^22^ judgements are made on an evaluation of critical and non-critical weaknesses. High: zero or one non-critical weakness; moderate: more than one non-critical weakness; low: one critical flaw without non-critical weaknesses; critically low: more than one critical flaw, with or without non-critical weaknesses.

**Table 3:** Quality evaluation for included reviews (Adapted Algorithm for GRADE,^18^ AMSTAR-2,^22^ risk of bias, heterogeneity, generalisability)

	AMSTAR-2							
Citation	Registered protocol	Comprehensive search strategy	Duplicate extraction	Total number of participants Pooled meta-analysis	RoB (bias and trial quality)	Heterogeneity evaluated	AMSTAR ranking	Level of evidence/and generalisability factors
Witt^21^	Yes	Yes	Yes	CBT versus TAU post intervention: Repeat self-harm 4 trials, *n* = 238; 6-month follow-up 9 trials, *n* = 2458; 12 months: 9 trials, *n* = 2458; CBT versus TAU or alternative therapy, frequency of self-harm by post intervention: *n* = 659; Emotion-regulation psychotherapy (ERP) versus TAU: 2 trials, *n* = 83. MBT versus TAU reducing repeat self-harm by 18 months and the frequency of self-harm: one trial, *n* = 63.	CROB: Most trials rated as having some or high risk of bias (84.2%). Main concerns included missing outcome data, selective reporting, outcome measurement. GRADE: CBT: low certainty evidence; DBT: very low certainty evidence; ERP: Moderate certainty; MBT: high certainty (1 trial).	Yes (low)	High	Downgraded 1 for trial quality (moderate evidence based on trial quality). Interventions delivered in in-patient or out-patient settings were eligible for inclusion. Only 6/76 trials were based in mental health in-patient settings. Population: patients with a recent presentation for self-harm, aged 18 or over.
Hawton^28^	Yes	Yes	Yes	CBT versus TAU self-harm repetition by 6 months: *n* = 1317; CBT versus TAU 12 months: *n* = 2232; suicide final follow-up: 2354; DBT: self-harm repetition post; intervention: 173; DBT: self-harm repetition *n* = 12 months: *n* = 77; DBT: suicide: *n* = 317.	CROB: Risks included performance bias. Quality of evidence was moderate to very low, biases typically related to blinding, which is challenging in psychosocial interventions.	Yes (ranged from low to 51%)	High	Downgraded 1 for trial quality (moderate evidence based on trial quality). Intervention delivered in in-patient or out-patient settings were eligible for inclusion. Most this evidence is derived from cross-setting interventions, and some trials excluded in-patient populations. Approximately 4/26 studies were based in mental health in-patient settings. Population: patients with a recent presentation for self-harm aged 18 or over.
Hou^29^	No	Yes	Yes	Insufficient information	CROB: 5 studies classified as high risk, predominately due to incomplete outcome data or blinding of outcome assessment.	Yes (Suicide: *I*^*2*^ = 17%, attempted suicide; *I*^*2*^ = 52%)	Critically low	Downgraded 1 for trial quality; Downgraded 1 for AMSTAR (2 downgrades) (moderate evidence based on trial quality). Mixed settings, with 3/16 in-patient settings. Others included out-patient, military installations, hospitals, emergency departments, crisis centre, primary care. Reported results for adults and adolescents together. Population: patients discharged from in-patient settings, those with recent suicide attempt or self-harm, young people at risk of suicide, military staff, prisoners, staff, older adults, students, middle aged men.
Fox^25^	No	Yes	Yes	Reported effect sizes number (overall: *n* = 1186, suicide: *n* = 159, suicide attempt: *n* = 209. non-suicidal self-injury: *n* = 46 self-harm: *n* = 73) extracted from Table 1.^25^	Quality Assessment Tool for Quantitative Studies. Low for publication bias. More than half of the effect sizes (57.58%) were associated with weak study quality. More than a quarter of the effect sizes (36.21%) were from studies with moderate quality, with only 6.22% of the effect sizes from studies with strong quality. Bias included, selection bias, blinding).	Yes (low)	Critically low	Downgraded 1 (AMSTAR): Downgraded 1 for trial quality (2 downgrades) (moderate evidence based on trial quality). Wide range of mixed settings and included studies. Approximately, 7/591 included studies were based in mental health in-patient settings. Mixed ages and population. Reported results for adults and adolescents together.
Yiu^31^	Yes	Yes	Yes	Meta-analysis	CROB 2: All studies: high risk of bias for blinding, several studies had high risk of bias for missing outcome data and selective reporting.	Yes (low)	Critically low	Downgraded 1 for trial quality (1 downgrade). Restricted to interventions adapted for psychiatric in-patients, based on 10 trials (moderate evidence based on trial quality).
Sobanski^30^	No	Yes	No	CBT: *n* = 961 Problem-solving: *n* = 474 Psychodynamic: *n* = 253 DBT: *n* = 256	CROB: Potential for publication bias and other biases across studies (e.g. blinding, incomplete outcome data, detection bias and other bias).	Yes (low-moderate)	Critically low	Downgraded 1 for trial quality; Downgraded 2 for AMSTAR (3 downgrades) (low evidence) Mixed settings, with approximately 4/ 18 trials based in mental health in-patient settings. Settings included In-patient and out-patient, community, emergency department. Population: adults aged 18 or over, with history of suicide attempts. Self-harm without intent was excluded. Mixed psychiatric diagnoses, patient populations (patients with suicidal behaviour during the last week, active-duty soldiers, people with recent suicide attempts, college students, people presenting to an emergency department, people with current major depressive episode reporting suicidal ideation or attempts.
DeCou^36^	No	Yes	No	*N* = 784 (DBT)	Not reported.	Yes (low)	Critically low	2 downgrades (AMSTAR); 1 downgrade (bias) Downgraded 1 for trial quality (low evidence). Cross-setting interventions, 3/18 based in mental health in-patient settings. Mix of in-patient and out-patient settings, populations and age. Reported results for adults and adolescents together. Population: Mostly in-patients and out-patients diagnosed with borderline personality disorder, bipolar disorder or people hospitalised for a suicide related event.

Narrative reviews (adapted GRADE is not applicable to narrative reviews)								
Citation	Registered protocol	Comprehensive search strategy	Duplicate extraction	Total number of participants Pooled meta-analysis	RoB (Bias and trial quality)	Heterogeneity evaluated	AMSTAR ranking	Generalisability factors
Wand^35^	No	Yes	Yes	Narrative	Quantitative research: Alberta Heritage Foundation for Medical Research Standard Quality Assessment Criteria. Quality studies varied widely; only six studies rated as high-quality. Underpowered, potential for Type 1 error. Lack of blinding for outcome ratings. Loss to follow-up not discussed.	Heterogeneity discussed in terms of study design, interventions, and outcomes.	Low	Settings included in-patient and out-patient hospital to community psychiatric care; 1/20 included post discharge from in-patient hospitals, 2/20 studies evaluated suicide as an outcome. Both evaluated a comprehensive and intensive older-age aftercare programme in Hong Kong, that included specialist aftercare for older adults that attempted suicide (case management, integrated follow-up with primary care).
Rozek^33^	No	Yes	Yes	Narrative	Effective Public Health Practice Project quality assessment tool. Overall, 10 studies were rated as strong, 17 studies rated as moderate, and six studies as weak. Bias included retention and recruitment, lack of control groups, inadequate power.	Heterogeneity discussed in discussion over study samples, designs, and outcome measures.	Low	Mixed settings, 2/33 included military mental health in-patient settings. Settings also included out-patients, mixed populations (e.g. history of self-harm, trauma, psychiatric diagnosis); complex variable interventions. Insufficient data reported for age. Population: people diagnosed with PTSD or experiencing trauma symptoms. Excluded people with non-suicidal self-injury.
Luxton^37^	No	Partial	No	Narrative	Not reported.	Not reported.	Critically low	Mixed settings, 1/11 included mental health in-patient settings. Most of the evidence comes from studies with patients discharged from emergency care. Population: people with history of suicide attempts, or treated for self-harm, or discharged from hospital, or treated an in-patient.
Mann^10^	No	Yes	No	Narrative	Not reported.	Heterogeneity discussed in the discussion for psychiatric illness, proportion of males, sample sizes, outcome measures, and complex interventions.	Critically low	Cross setting interventions, approximately 6/ 97 trials based in mental health in-patient settings. Wide variability in quality and quantity of data available, wide heterogeneity of study populations, interventions, proportion of high-risk patients (history of suicide attempt, age, ethnicity, men). Reported combined results of psychotherapy trials for adults and adolescents.
Donker^34^	No	Yes	Yes	Narrative	Jadad’s quality criteria. Most studies measured as adequate. Bias included retention and incomplete outcome data.	Not reported.	Critically low	Mixed settings, 2/10 trials based in mental health in-patient settings. Other settings included out-patient settings. Age ranged from 15 to 65. Reported combined results for adults and adolescents. Population: people diagnosed with schizophrenia spectrum/psychotic disorders.
Nawaz^32^	Yes	Yes	Yes	Narrative	Effective Public Health Practice Project quality assessment tool. Only 1 study had strong quality rating, five were moderate and 17 were weak. Bias included bias and masking.	Discussion of heterogeneity of study design, interventions, and outcome measures as justification for narrative analysis.	Critically low	Most studies used pre–post designs, with small sample sizes, several without controls. Settings and population: restricted to in-patient settings, no restrictions on psychiatric condition. Reported combined results for adults and adolescents.

Adapted GRADE scoring: reviews are rated as providing a high level of evidence if they have received zero downgrades, moderate if one or two downgrades, low if three or four downgrades and very low if the review receives five or six downgrades (not applicable for narrative reviews).

CT, controlled trials; CCT, controlled cohort studies; CBT, cognitive behaviour therapy; CAT, cognitive analytic therapy; CPDpd, Cognitive behaviour therapy adapted for people who have received a personality disorder diagnosis; CROB, Cochrane Risk of Bias tool; EUC, enhanced usual care; MBT, mentalisation-based therapy; DBT, dialectical behaviour therapy; RCT, randomised controlled trials; ITS, interrupted times series designs; Obs., observational study design; PP, pre–post; QED, quasi-experimental designs; RoB, risk of bias; TAU, treatment as usual.; CROB, Cochrane Risk of Bias tool.

### Characteristics of included studies

Characteristics of the included reviews (e.g. including interventions, outcomes, comparators, bias, heterogeneity) are presented in Tables 3 and 4 and are summarised here. Additional information is presented in the Supplementary material. In total, 13 reviews evaluated interventions that included in-patient mental health settings. The systematic reviews were published between 2013 and 2022. The 949 primary studies were published between 1970 and 2021, inclusive, with an approximate total of over 160 000 participants studied in the reported primary research. Overall, most primary studies were conducted in Western Europe and North America. Five reviews reported data for adults,^21,28,30,31,35^ six reported composite data for adults and adolescents^10,25,29,32,34,36^ and two reviews had insufficient reporting for age.^33,37^ Wand et al^35^ evaluated interventions for older adults. The percentage of female participants in the included primary studies ranged from 6 to 98%, and 6/13 reviews had insufficient reporting for gender. Seven reviews conducted meta-analyses,^21,25,28–31,36^ and six narratively summarised the data.^32–35,37^ Most reviews evaluated cross-setting interventions that included in-patient settings and two reviews specifically focused on in-patient settings.^32,33^ Six reviews evaluated post-discharge interventions as part of their overall review,^10,21,29,30,35,36,38^ six evaluated ward-based interventions^21,30,33,38^ and three had insufficient reporting as regards the timing of the intervention.^25,34,36^

**Table 4:** Study characteristics for included reviews (e.g. included study dates, age, gender intervention details, controls, follow-up, designs, outcomes)

Citation	Date	Studies (*N*)	*N*	Country	Age	Gender %F	Population	Settings
Hou^29^	1976–2021	16	5338		Mean 32.2; s.d. = 12.8		Patients discharged from hospital settings, patients with recent suicide attempt or self-harm, or at elevated risk of suicide.	Mixed settings (e.g. out-patient, military clinics, hospitals, emergency departments, crisis centre, primary care). No description of wards or in-patient settings.
Witt^21^	1977–2020	76	21 414	Europe, Oceania, N America, Middle East, Asia	Mean 31.88; s.d. = 11.7	61	Patients with a recent presentation for self-harm, aged 18 or over.	In-patient or out-patient settings. Six trials included in-patient settings, 4/6 received treatment and out-patient follow-up, two studies evaluated treatment during in-patient stays. No ward-specific details.
Hawton^28^	1978–2014	26	8480	Europe, N America, Middle East, Asia, Oceania	Mean 25.5; s.d. = 15.7	71	Patients with a recent presentation for self-harm, aged 18 or over.	In-patient or out-patient settings. Most of this data pertains to emergency department and out-patient treatment. Psychiatric in-patients excluded from several studies. No description of wards.
Sobanski^30^	1990–2020	18	1990	Undefined in review	Mean 20.4; s.d. = 0.76 to 44.8; s.d. = 16.4	16–90	Adults aged 18 or over, with history of suicide attempts, mixed psychiatric diagnosis.	In-patient and out-patient, community, emergency department. No description of wards.
Fox^25^	1970–2020	591	1186	Europe, N America, Middle East, Asia, Oceania	Mean 33; s.d. = 13.4	62	Population as percentage of effect sizes (*n* = 3,458) patients with psychopathology 60.3%; history of self-injurious behaviour 28.2%; general population samples 11.5%.	Cross-setting interventions including emergency departments, in-patient and out-patients. No descriptions of wards.
DeCou^36^	1999–2016	18	987		Mean 31; s.d. = 7.3		In-patients and out-patients diagnosed with borderline personality disorder, bipolar disorder, or people hospitalised for a suicide attempt.	Cross-setting interventions, mostly in out-patient settings. No description of ward settings.
Yiu^31^	1981–2020	10	976	USA/UK	Mean 36.3; s.d. = 6.7	6–98	Psychiatric in-patients, any mental illness diagnosis.	All adult in-patient settings; 9/10 studies based in an in-patient psychiatric unit; 1/10 in a community crisis stabilisation unit. No description of ward settings.

Citation	Interventions as defined by authors	Controls	Follow-up	Designs	Synthesis	Outcomes
Hou^29^	Social support interventions to prevent suicide (postal, text, face-to-face, email). Social support interventions were defined as having at least one intervention component that promoted social support/ connectedness, or decreased social isolation/feelings of loneliness (post-discharge interventions).	TAU, waitlist	Three months to 5 years	RCT	Meta-analysis (interventions pooled).	Suicide attempts, suicide.
Witt^21^	Psychosocial interventions for self-harm defined as individual or group-based psychological therapy, that may vary in delivery, location of treatment, frequency, and intensity (e.g. CBT, problem-solving therapy, DBT, MBT, emotion regulation psychotherapy, psychodynamic psychotherapy, case management, structured general practitioner, brief emergency department-based interventions, remote contact interventions, provision of follow-up and support, multimodal interventions, mixed interventions). Categorisation of interventions based on trials and expert consensus (includes ward and post-discharge interventions).	TAU, or other (None, EUC)	0, 6, 12 months post-treatment	RCT	Meta-analysis. Interventions and outcomes analysed separately.	Repeated self-harm, attempted suicide, suicide.
Hawton^28^	Effectiveness of suicide prevention interventions (CBT, brief contact interventions, outreach, problem-solving, adherence, continuity programme, safety plans, postcards, telephone follow-up, brief CBT, attempted suicide short intervention programme, DBT). Categorisation of interventions based on trials and expert consensus (includes ward and post-discharge interventions).	TAU, lower intensity active control	Six,12, to 24 months follow-up	RCT	Meta-analysis. Interventions and outcomes analysed separately.	Repeated self-harm, attempted suicide, suicide.
Sobanski^30^	Psychotherapeutic interventions based on psychological theories and psychological science. The purpose of treatment was to assess and modify behaviours, cognitions, or emotion to reduce suicide re-attempts (e.g. CBT, DBT, psychodynamic psychotherapy problem-solving). Ward and post-discharge-based interventions (e.g. PACT, CBT).	TAU	3 to 24 months	RCT	Meta-analysis. Interventions analysed together and separately for CBT, problem-solving therapy, psychodynamic psychotherapy, and DBT.	Suicide attempts/suicide. Composite. Excluded: Self-harm.
Fox^25^	Interventions for suicide and self-injury. Percentage of effect sizes: CT, CBT 11.7%; DBT 7%, combinations of therapy and meds 6.3%; checking-in programmes 2.5%; problem-solving therapy 1.6%; safety planning and/or means restriction 1.47%; psychoanalysis 0.93%; and in-patient hospitalisation 0.12%. Insufficient information on timing of intervention.	Active control, placebo, non, TAU	Pre–post treatment, first, last, all follow-ups	RCT	Meta-analyses. Interventions pooled and analysed separately in moderator analyses.	Suicide, suicide attempts, NSSI, self-harm (composite and separate in meta-analyses).
DeCou^36^	Dialectical behaviour therapy, defined as treating emotional dysregulation, via providing dialectical strategies, core strategies (validation and problem-solving), CBT strategies, communication strategies, case management and structural strategies. Insufficient information on timing of intervention.	TAU, waitlist, active control	Two weeks to 12 months	RCT	Meta-analysis.	Suicidal behaviours, self-harm (composite).
Yiu^31^	Psychosocial interventions, defined as non-pharmacological interventions targeting psychological or social factors that can reduce suicide or self-harm in people with mental health conditions (CBT, DBT, diary entries, Insight-orientated therapy, cognitive restructuring, peer support for suicide prevention, creating coping skills training) (Ward-based interventions).	TAU, enhanced TAU, active control	1 month to 6 months	RCT	Meta-analysis. Separate analyses for suicide attempts and other outcomes (Suicidal ideation, depression, hopelessness). Interventions pooled together.	Suicidality (main outcome), self-harm (no studies had self-harm as an outcome), suicide attempts.

Narrative reviews								
Citation	Studies (date)	Studies (*N*)	*N*	Country	Age	Gender %F	Population	Settings
Nawaz^32^	1993–2018	23	2402	USA, Europe, S Asia.	12–18 (adolescent wards) 16–70 (adult wards, 7 studies did not report the age of patients)	75	Psychiatric in-patients receiving interventions for self-harm. No restrictions on age, diagnosis.	All in-patient settings (forensic, psychiatric intensive care units (PICUs) and adolescent wards. Ward details: *N* = 62 in-patient wards, that included 41 acute psychiatric wards, 6 forensic, 7 child and adolescent, 4 PICU, and 3 triage/assessment units.
Rozek^33^	2011–2021	33					Patients with co-occurring suicidal thoughts and behaviours and post-traumatic stress disorder.	Mixed settings, mostly military, but also in-patient and out-patient. No ward-specific information.
Wand^35^	2010–2020	20	101394	Europe, Asia	Mean = 75.5; s.d. = 6.9	57	Older adults who have self-harmed.	Cross-setting interventions (e.g. in-patient and out-patient hospital to community psychiatric care; post-discharge from in-patient hospitals).
Luxton^36^	1975–2010	11	8598	N America, Oceania, Europe			People with history of suicide attempts, self-harm, or who were discharged from hospital, or treated as an in-patient.	Mixed settings. Mostly patients discharged from emergency care. No description of wards.
Donker^34^	2002–2010	10	3574	Europe	Range: 15–25, 16+, and 18+		People diagnosed with schizophrenia spectrum/psychotic disorders.	Cross-setting interventions: (in-patient, out-patients). No description of wards.
Mann^10^	2005–2019	4	3363	N America, Europe, SE Asia, E Asia	Range: adolescents 12–18, adults: 18–75	55–57	People at risk of suicide.	Cross-setting interventions (in-patient, out-patient, emergency departments, community). No description of wards.

Narrative reviews						
Citation	Interventions as defined by authors	Controls	Follow-up	Designs	Synthesis	Population
Nawaz^32^	Interventions were included if they reported any impact on self-harm (DBT, problem-solving therapy, skills training, unified protocol, phone-based positive psychology, post-admission cognitive therapy, ward-based interventions, mixed interventions). Studies were grouped into therapeutic, ward-based, or mixed interventions. **Therapeutic interventions:** DBT adapted for in-patient settings (*n* = 8); 5 used an adapted in-patient DBT for patients who received a diagnosis of emotional personality disorder, and a history of self-harm; 3 studies used adapted DBT for adolescent in-patients. Other therapeutic interventions included: ‘Skills to enhance positivity (STEPs)’ (*n* = 1); systems training for emotional predictability and problem-solving (STEPPS) for people who received a diagnosis of personality disorder (*n* = 1) for adolescents, respectively. Problem-solving therapy (*n* = 2) post-admission cognitive behaviour therapy (*n* = 1), phoned-based positive psychology (*n* = 1). **Ward-based interventions**: **Safewards (*n* = 3) and staff training (*n* = 3):** Safewards intervention comprised 10 interventions focused on patient-centred care and behaviour standards for patients and staff to reduce containment rates. This intervention was used in three studies, including one study based on forensic wards. **Training interventions (*n* = 3)** included: (a) employing clinical experts to work with ward staff 3 days per week to support low conflict, low-containment therapy-based nursing; (b) alternatives to constant observation using 17-point behavioural checklist; (c) collaborative problem-solving, to compare behavioural outcomes and staff perceptions during pre–post training phases for a 5-year study**. Mixed interventions (*n* = 2)** included: (a) zonal nursing and co-designing therapeutic days, including recreational, therapeutic, and physical activities; and (b) provision of a regular ‘twilight’ shift, and a structured evening activities programme.		1 month to 5 years	RCT, CT, ITS, PP	Narrative	Self-harm (narrative)
Rozek^33^	Evidence-based PTSD treatments that address co-occurring suicidal thoughts and behaviours and post-traumatic stress disorder in evidence-based psychotherapies for adults: (e.g. DBT, brief cognitive behaviour therapy, post-admission cognitive therapy for the prevention of suicide (PACT) interpersonal psychotherapy, problem solving). **Ward interventions** **Suicide-specific treatments** included protocols that targeted suicide or risk of suicidal behaviour and ideation: BCBT: Brief cognitive behaviour therapy. PACT: post-admission cognitive therapy for the prevention of suicide. DBT: dialectical behaviour therapy (ward-based interventions).	TAU, waitlist, enhanced care			Narrative	Suicidal behaviours (narrative) Excluded non-suicidal self-injury
Wand^35^	Evidence-based aftercare for older adults following self-harm. Social support interventions to prevent suicide (postal, text, face-to-face, email) (post-discharge intervention in specialist older adult services).	TAU without follow-up usual care	24 months	Historical observational cohort	Narrative	Suicide, suicide re-attempts
Luxton^36^	Post-discharge suicide prevention interventions that involve follow-up. Follow-up interventions included at least one form of pre-planned follow-up contact (letters, postcards, electronic), phone calls, in-person visits, which were initiated by the care providers and were not part of a larger psychotherapy trial (post-discharge intervention).		1 month to 5 years	PP, Obs.	Narrative	Suicide, suicide attempts, self-harm (narrative)
Donker^34^	Psychosocial interventions defined as any intervention that provides psychoeducation or psychotherapy and delivered through any format. Interventions included integrated motivational interviewing plus CBT, psychoeducation, psychotherapy, case management, counselling, or community treatment). Insufficient information on timing.	TAU	10 weeks to 18 months	RCT/CT	Narrative	Suicide, attempted suicide, self-harm
Mann^10^	Suicide prevention strategies included psychotherapy interventions (e.g. Cognitive therapy, skills-based treatment, CBT for PD, DBT, CBT, MBT, CBT, DBT, CAMS, Acceptance and commitment therapy, mood-regulation focused cognitive therapy, Post-admission cognitive therapy). Contact or active outreach (e.g. telephone contacts, postcards, brief contact interventions, rapid outreach, assertive outreach, crisis response plans), internet-based interventions. System, ward, and post-discharge interventions, but insufficient reporting regarding timing.	TAU	8 months to 24 months	RCT Obs, QED	Narrative	Suicide, self-harm, suicide re-attempts

CT, controlled trials; CBT, cognitive behaviour therapy; EUC, enhanced usual care; MBT, mentalisation-based therapy; DBT, dialectical behaviour therapy; RCT, randomised controlled trials; ITS, interrupted times series designs; Obs, observational study design; PP, pre–post; QED, quasi-experimental designs; TAU, treatment as usual; NSSI, non-suicidal self-injury; PD, patients diagnosed with personality disorder; CAMS, collaborative assessment and management of suicidality.

### Quantitative reviews

#### Cognitive–behavioural therapy (CBT)

In an updated Cochrane review of psychosocial interventionsns for self-harm,^11,28^ Witt et al^21^ found that CBT reduced self-harm repetition compared with treatment as usual by post-intervention (odds ratio 0.35, 95% CI [0.12 to 1.02]; *N* = 238; *k* = 4; *I*^*2*^ = 0%), 6-month (odds ratio 0.52, 95% CI [0.38 to 0.70]; *N* = 1260; *k* = 12; *I*^*2*^ = 2%) and 12-month follow up (odds ratio 0.81, 95% CI [0.66 to 0.99]; *N* = 2458; *k* = 9; *I*^*2*^ = 0%). The evidence suggested that CBT reduced the frequency of self-harm repetition at six- (mean difference −0.71, 95% CI [−1.32 to −0.11]; *N* = 118; *k* = 4; *I*^*2*^ = 0%) and 12-month follow-up (mean difference 1.18, s.d. = 4.22, *n* = 40 versus mean difference 4.58; s.d. 8.37; *n* = 33; mean difference –3.40, 95% CI [−6.54 to −0.26]; *N* = 73; *k* = 1; *I*^*2*^ not applicable), but not the post-intervention assessment. Using the GRADE criteria,^19^ Witt et al^21^ rated the quality of evidence as ‘low certainty’.

Sobanski et al^30^ found that pooled interventions for patients who attempted suicide resulted in significantly fewer episodes of suicidal behaviour compared with controls (relative risk 0.66; 95% CI [0.48, 0.90]; *Z* = 2.63, *p* < 0.01; odds ratio 0.56, 95% CI [0.36–0.84], *p* < 0.01, *k* = 18, *I*^2^ = 51%). In separate intervention analyses, Sobanksi et al^30^ found significant treatment effects for CBT compared with treatment as usual (relative risk 0.66; 95% CI [0.48–0.90]; *Z* = 2.61, *p* = 0.009; odds ratio 0.53, 95% CI [0.34–0.83]; *k* = 10, *p* = 0.005, *I*^2^ = 28%) and psychodynamic interventions (mentalisation-based therapy (MBT), brief psychodynamic interpersonal therapy) in reducing suicide re-attempts frequencies (relative risk 0.21; 95% CI [0.08–0.57]; *Z* = 3.08, *p* = 0.002; odds ratio 0.17, 95% CI [0.06–0.45]; *k* = 2, *p* < 0.0004, *I*^2^ = 30%). However, treatment effects for CBT were only significant for longer follow-up for (>12 months) (relative risk 0.60, *Z* = 2.38, *p* = 0.02). However, in a meta-analysis that evaluated pooled interventions (e.g. post-admission CBT, dialectical beahviour therapy (DBT), insight-oriented therapy, gratitude diaries) based in psychiatric in-patient settings, Yiu et al^31^ found no significant differences between treatment and control conditions for suicide attempts (relative risk 0.92; 95% CI [0.41–2.06]; *Z* = 0.18, *k* = 10, *p* = 0.86, *I*^2^ = 0%).

#### Dialectical behaviour therapy (DBT)

Evidence from three meta-analyses indicated that DBT may be effective in reducing the frequency of self-harm.^21,25,28^ Witt et al^21^ found evidence of beneficial treatment effects for DBT in reducing the frequency of repeated self-harm by post-intervention follow-up (mean difference −5.00, 95% CI [−8.92 to −1.08]; *N* = 659; *k* = 7; *I*^*2*^ = 49%). Using a composite outcome that included suicide attempts, non-suicidal self-injury, self-harm and suicide, DeCou et al^36^ found positive treatment effects for DBT in reducing ‘self-directed violence’ compared with controls (weighted mean effect size, *d* = −0.324, 95% CI [−0.471 to −0.176], *k* = 15, *I*^*2*^ = 45.48%).^36^

#### Other interventions (MBT, emotion-based regulation psychotherapy, social support interventions)

Based on one trial rated as providing high certainty evidence, Witt et al^21^ found evidence to suggest that mentalisation-based therapy may reduce self-harm repetition (18/71 versus 31/63; odds ratio 0.35, 95% CI [0.17 to 0.73]; *N* = 134; *k* = 1; *I*^*2*^ = not applicable) and the frequency of repetition (mean difference 0.38, s.d. = 0.38, *n* = 71 versus mean 1.66, s.d. = 2.87, *N* = 63; mean difference -1.28, 95% CI [−2.01 to −0.55]; *N* = 134; *k* = 1; *I*^*2*^ = not applicable). Based on moderate certainty evidence (GRADE)^19^, Witt et al^21^ found positive treatment effects from group-based emotional regulation psychotherapy for reducing repeat self-harm, but not for the frequency of repetition (odds ratio 0.34, 95% CI [0.13 to 0.88]; *N* = 83; *k* = 2; *I*^*2*^ = 0%).

Hou et al^29^ found evidence to suggest that social support interventions, defined as having at least one intervention component that promoted social support/connectedness, or decreased social isolation/feelings of loneliness reduced deaths by suicide (pooled relative risk 0.48, 95% CI [0.27 to 0.85], *k* = 10, *p* = 0.01). Social support interventions had greater benefit for reducing suicide, when delivered face-to-face, for people who had attempted suicide, but not for other delivery methods or populations (relative risk 0.24, 95% CI [0.10 to 0.58]).^29^

### Narrative reviews

#### CBT and DBT

Consistent with the meta-analyses,^21,28^ the results from two narrative reviews indicated beneficial effects for CBT in reducing suicide attempts.^10,33^ Rozek et al^33^ evaluated psychotherapies to address co-occurring suicidal thoughts and behaviours and post-traumatic stress disorder (PTSD). Suicide-specific treatments in this review,^33^ significantly reduced suicidal behaviour outcomes and PTSD symptoms. Three narrative reviews suggested that DBT was beneficial in reducing self-harm and suicide attempts.^10,32,33^ In their evaluation of interventions to reduce self-harm on in-patient wards, Nawaz et al^32^ found that DBT was the most frequently implemented and effective intervention to reduce self-harm. Additional supportive evidence was ascertained for the systems training emotional predictability and problem-solving therapy (STEPPs) intervention in reducing hospital admissions for self-harm for patients diagnosed with borderline personality disorder.^32^

#### Post-discharge follow-up contacts

Luxton et al^37^ indicated that repeated follow-up contact (postcards/ telephone contact) for patients discharged from hospital may reduce repeat suicide attempts (3 studies) and suicide (2 studies). Other studies in this review reported inconclusive results or did not demonstrate any preventative effects. In their review of evidence-based interventions for suicide prevention, Mann et al^10^ also suggested that post-discharge follow-up contact (e.g. brief contact, enhanced follow-up, caring texts) reduced suicidal behaviour. Wand et al^35^ suggested a comprehensive aftercare programme for older adults may be beneficial in reducing suicide. However, the strength of evidence was poor, with significant methodological limitations, heterogeneity and small absolute risk reductions.^35^

#### System, staff training or ward-level interventions

Mann et al^10^ suggested system-level changes may be effective in reducing suicide via evidence from two UK studies evaluating the implementation of evidence-based recommendations (e.g. improved depression management, low staff turnover, continuity of care) in mental health services. Nawaz et al^32^ found evidence to suggest that mixed interventions that combine therapeutic and ward-based approaches significantly reduced self-harm (2 studies). Ward-based interventions to prevent self-harm were inconclusive, with three studies showing reductions in self-harm, and three that did not. Staff training that included the provision of additional nurses on two acute wards, assistance with implementation of changes according to a model of conflict and containment (one study), and problem-solving training (one study), significantly reduced self-harm on in-patient wards. Nawaz et al^32^ found evidence to suggest that combinations of a therapeutic approach and ward-based changes also reduced self-harm (2 studies).^32^ However, most of this evidence was based on weaker pre–post designs with small sample sizes, with complex poorly defined interventions.^32^

### Non-significant findings for interventions

Several reviews evaluated interventions, but did not find statistically significant treatment effects for reducing self-harm, attempted suicide, or suicide.^21,25,29–34^ These interventions are listed in Table 5.

**Table 5:** Non-significant treatment effects for interventions in included systematic reviews

Citation	Interventions	Outcome indicating non-significant treatment effects	Synthesis
Hawton;^28^ Witt;^21^ Fox^25^	Cognitive-behaviour therapy (CBT) versus treatment as usual (TAU).	Reducing self-harm repetition; deaths by suicide.	Meta-analysis
Hawton;^28^ Witt;^21^ Fox^25^	Group-based CBT-based interventions.	Self-harm repetition or frequency of repetition.	Meta-analysis
Hawton;^28^ Witt;^21^ Fox;^25^ Sobanksi^30^	Dialectical behaviour therapy (DBT) versus TAU.	Reducing repeat self-harm by post-intervention or 12-month follow-up; or deaths by suicide.	Meta-analysis
Hawton;^28^ Witt;^21^ Fox^25^	DBT-group-based skills training compared with standard DBT.	Suicide re-attempts, non-suicidal self-injury by the post-intervention period, frequency of self-harm by the 12-month follow-up.	Meta-analysis
Hawton;^28^ Witt^21^	Individual-based DBT versus standard DBT.	Suicide re-attempts.	Meta-analysis
Hawton;^28^ Witt^21^	Psychodynamic psychotherapy, interpersonal problem-solving therapy, continuity of care by same therapist, behaviour therapy, case management, intensive in-patient, and out-patient treatment; remote contacts, coping cards, telephone contact, provision of information and support.	Reducing repeat self-harm.	Meta-analysis
Hou^29^	Social support interventions.	Suicide attempts.	Meta-analysis
Yiu^31^	Combined: Post-admission cognitive therapy, behaviour therapy, nsight t-oriented therapy, gratitude journal.	Suicide attempts.	Meta-analysis
Nawaz;^32^ Sobanksi^30^	Problem solving, post-admission cognitive therapy, and phone-based positive psychology.	Self-harm rates.	Narrative
Donker^34^	CBT, DBT, motivational interviewing, supportive counselling compared with controls for patients with schizophrenia spectrum disorders and psychosis.	Self-harm, attempted suicide, or suicide between intervention groups and controls.	Narrative

#### Review overlap: fraction of evidence synthesised in two or more reviews

**Fig. 2:**
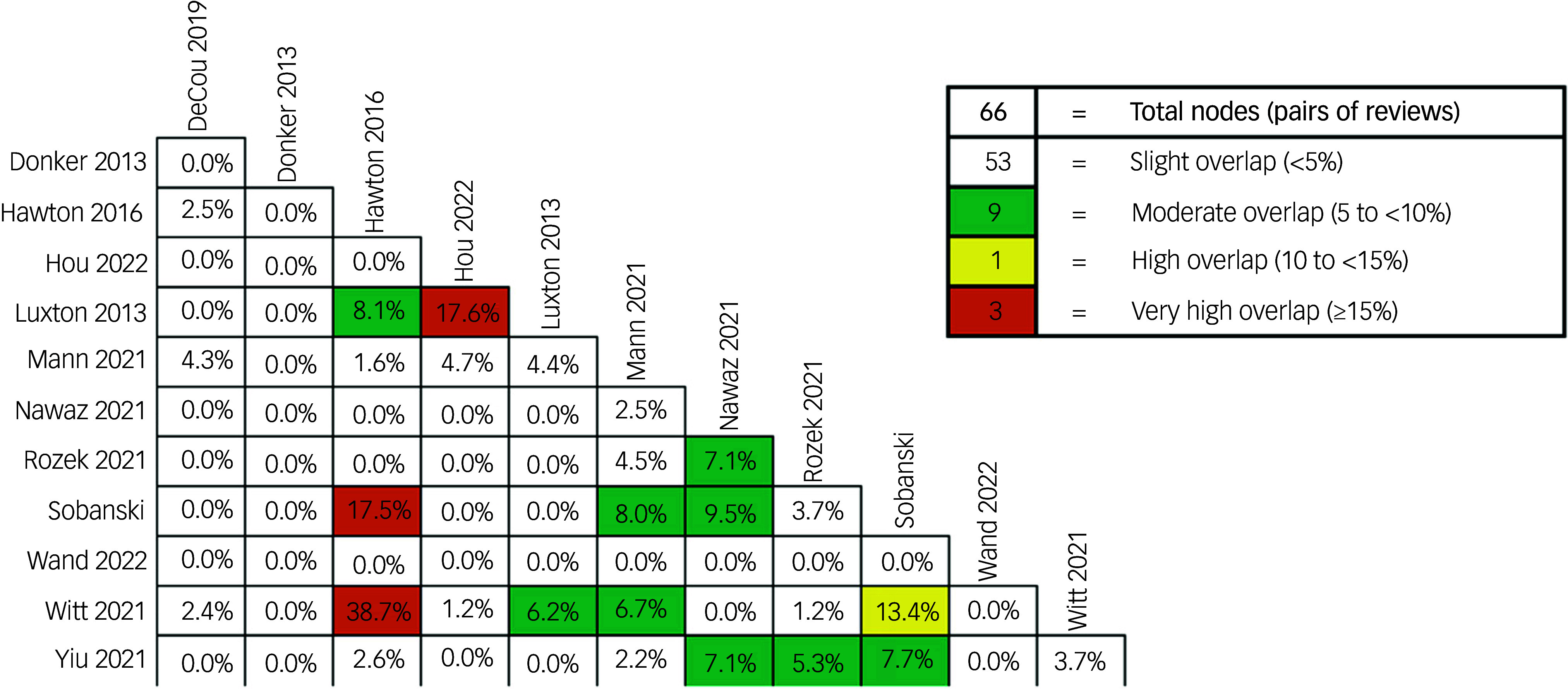
Heat map for primary study overlap analysis for citations for self-harm, suicide and suicidal behaviour.

Figure 2 presents the Graphical Representation of Overlap for OVErviews (GROOVE) heat map for the primary study overlap analysis.^23^ The Covered Area for the reviews was 12.4% and the Corrected Covered Area was 4.4%, indicating a slight degree of overlap for the overall review.^23^ Some pairs of reviews had ‘very high’, or ‘moderate’ overlap. For example, Sobanksi et al^30^ had very high overlap with Hawton et al^28^ and Luxton et al^37^ had high overlap with Hou et al^29^ As expected, Witt et al^21^ had high overlap with Hawton et al^28^ given this was an updated review of the work. Yiu et al^31^ had moderate overlap with Nawaz et al^32^ Rozek et al^33^ and Sobanski et al^30^

## Discussion

We synthesised evidence from 13 systematic reviews assessing the efficacy and/or the effectiveness of self-harm and suicide prevention interventions. Our aim was to evaluate the quality and relevance of this evidence for reducing the likelihood of self-harm and/or suicide in mental health in-patient settings. Overall, most quantitative and narrative reviews suggested support for CBT and DBT in reducing self-harm and suicide attempts, but evidence for reducing suicide deaths was limited. Narrative reviews highlighted promising interventions, including post-discharge follow-up, implementing evidence-based recommendations,^10^ ward-based changes (e.g. additional nurses, increased access to therapeutic activities) and staff training as part of broader interventions.^32^ However, their real-world applicability and effectiveness in mental health in-patient settings is less clear due to differences in patient populations, high heterogeneity in the synthesis of interventions and a lack of pragmatic trials and co-production.

CBT demonstrated efficacy in reducing repeat self-harm and suicide attempts, particularly with longer follow-up.^10,21,30^ DBT was associated with a reduction in the frequency of repeat self-harm, but not in reducing the likelihood of repetition.^10,21,25^ However, these findings should be interpreted cautiously, as only a small subset of primary studies included in these reviews originated from in-patient settings. For example, Witt et al^21^ included only six trials from in-patient settings. CBT may also be more suitable to community settings, whereas DBT could be more effective in reducing self-harm on in-patient wards.^32^ However, the evidence base for DBT includes relatively weak trials with specific populations.^21^ We know little of the effectiveness for adapted interventions as they are used in mental health in-patient settings.^32^

Other reviews had conflicting results on the effectiveness of CBT for reducing suicide attempts.^25,31,34^ Yiu et al^31^ found no statistically significant evidence supporting psychosocial interventions compared with treatment as usual in reducing suicide attempts for mental health in-patients, and no studies evaluating self-harm as an outcome. Lack of evidence for effectiveness and conflicting findings may be due to the review design (e.g. heterogeneous intervention types, duration, follow-up and comparator treatments). The review evidence also highlights the importance of tailoring interventions to clinical need. For example, one systematic review found no significant effect of CBT in preventing suicide attempts among patients diagnosed with schizophrenia spectrum disorders and psychosis.^34^ Evidence from another review suggested that brief CBT for suicide prevention may be effective in reducing co-occurring trauma symptoms and suicide attempts.^33^

### Quality of the evidence

Methodological limitations across both primary studies and systematic reviews emphasise the need for improved quality research and reporting. Only two reviews met ratings for ‘high quality’ according to the AMSTAR-2^22^ criteria.^21,28^ Only two reviews, led by the same team, evaluated the certainty of the evidence for clinical practice.^21,28^ Witt et al^21^ rated evidence quality as low to moderate for CBT-based psychotherapy, moderate for emotion-regulation therapy and very low quality for DBT versus treatment as usual.

### Strengths and limitations

Clinical demands, and the large volume of publications on self-harm and suicide prevention interventions, may reduce the likelihood of evidence translation into mental health in-patient settings. Our evidence synthesis of 13 systematic reviews highlights important evidence gaps that lay the foundation for future research. We provide detailed evidence evaluations and summaries to support knowledge mobilisation and translation to high-demand clinical practice. We did not conduct any meta-analyses, due to the high degree of heterogeneity among the included reviews and the potential for misleading conclusions. Restricting our analyses to meta-analyses of controlled trials, may have resulted in the exclusion of potentially promising system-level interventions. However, we provide important statistical parameters, detailed results and methodological details in our results. We focused on self-harm and suicide because they are key patient safety outcomes.^38^ Other outcomes, including quality of life, functioning and mental health symptoms are also important,^39^ but were beyond the scope of this umbrella review.

Although we used a published search strategy^6^ and broad approach, we may have missed some published reviews, including those published in countries where English is not widely used. We utilised robust methodological assessments, including AMSTAR-2^22^ and the adapted GRADE algorithm,^18^ and provide a transparent detailed evaluation of the evidence base to inform clinical practice. However, umbrella reviews are subject to multiple sources of bias, variable reporting and heterogeneity in primary studies and reviews. Included studies varied greatly in methodological robustness, and ranged from observational designs to randomised controlled trials, with many subject to bias (e.g. attrition, reporting). Nearly half of the reviews lacked sufficient gender-related data, while other reviews typically reported binary data (male/female). Reporting for other protected characteristics, such as ethnicity, physical disabilities, neurodivergence, as well as socio-economic position, was largely absent, possibly due to deficiencies in the primary study reporting.

The reporting quality for age varied across reviews, which is a limitation of the evidence base. Several reviews reported composite data combining children/adolescents with adults,^25,29,34,36^ while others reported age inadequately.^33,36^ Descriptions of ward settings were inadequately reported in most systematic reviews, limiting the ability to assess the effectiveness of interventions in specific in-patient care settings (e.g. intensive versus acute). To optimise the evaluation of interventions in mental health care, future primary research and systematic reviews should provide granular and detailed information on ward type and in-patient setting (e.g. acute wards, psychiatric intensive care units, forensic wards). While we excluded research based in custodial criminal justice settings, one review^(32)^ reported interventions that were evaluated in forensic wards. We do not know if these wards were included in other reviews due to poor reporting. These systematic limitations in the evidence base highlight key biases that should inform future research.

There was no patient and public involvement and engagement reported in reviews or primary studies, which is a substantial limitation. However, we integrated lived experience perspectives throughout this umbrella review process, ensuring experiential evaluation and relevance to real-world clinical practice. Our research team consisted of people with lived experience and a diverse, multidisciplinary group of health services researchers, clinicians and methodologists, which enriched our evidence synthesis and our interpretation of it. We excluded reviews of qualitative research, which is a limitation. However, our aim was to summarise the effectiveness of interventions, based on systematic review evidence. We have completed a lived experience commentary alongside this review, to enrich our summary of quantitative reviews.

### Comparisons with other research

Our findings are consistent with previous research emphasising the need for better quality intervention trials.^21^ Our synthesis supports conclusions that CBT-based interventions have the strongest evidence base for reducing repeat self-harm, while DBT may be more effective for decreasing the frequency of self-harm repetition.^9,40–41^ Our conclusions align with those reported from other reviews.^9,21^ Although psychosocial interventions show promise in reducing self-harm and suicide, methodological limitations in primary studies and insufficient inclusion of lived-experience involvement weaken the strength of the evidence base.

Research on developing psychological interventions for in-patient wards and to prevent self-harm and suicide is rapidly expanding.^42^ Recent evidence from a randomised controlled trial with 200 mental health in-patient participants, found evidence to suggest that adding brief CBT to treatment as usual significantly reduced post-discharge 6-month suicide reattempts.^43^ Consistent with Rozek et al,^33^ a high-quality review^44^ found that both direct and indirect suicide prevention interventions reduced suicide attempts. Hajek Gross et al^45^ found no significant effect for mentalisation therapy in reducing self-harm repetition compared with controls, which contrasts with the preliminary evidence cited in Witt et al.^21^ Pre–post studies in this review suggested a reduction in self-harm frequency, with longer treatment durations yielding greater effects.^45^ Future, co-designed, qualitative trial research in this area may provide important insights into intervention development for self-harm and suicide prevention in mental health in-patient settings.

### Clinical implications

In-patient mental health settings are a key setting for suicide prevention.^4^ Efforts to reduce self-harm and suicide in this setting, have predominantly focused on environmental adaptations, including ligature removal or restrictive practice.^9^ In the UK, in-patient suicide rates have remained static since 2016,^4^ highlighting the urgent need to consider interventions that may improve patient safety in this setting. Access to therapeutic interventions and care may improve patient safety and experiences, and reduce suicide rates. However, evidence from this umbrella review suggests an urgent need to develop self-harm and suicide prevention interventions that are feasible and acceptable for mental health in-patient settings. Implementation barriers include poor fidelity to interventions, inadequate staff training and the challenge of adapting interventions to high-demand ward environments and acute patient crises.^13,46,47^.

Interventions may be more widely implemented if developed collaboratively with staff and patients as part of quality improvement efforts.^48^,^49^ Embedding lived/living experience perspectives throughout all stages, from study design to implementation and evaluation, may enhance intervention relevance and acceptability. Weak evidence for psychosocial interventions does not necessarily indicate a lack of clinical benefit, but may reflect the omission of patient-centred outcomes. Reductions in self-harm may not always align with patient priorities, and interventions might provide benefits in broader areas, including general functioning, social participation and engagement with services.^39^

As the intervention evidence-base continues to develop for in-patient settings, immediate steps can be taken to reduce self-harm and suicide and improve patient experience.^49^ The UK National Health Service has introduced co-produced standards of care for in-patient mental healthcare,^49^ emphasising equity, trauma-informed practice, autism-informed approaches and cultural competence (see Table 6 for a summary of the Culture of Care Standards core commitments). Future psychosocial or system-level interventions should be compassionate, patient-centred and aligned to these standards to ensure clinical relevance.

**Table 6:** The Culture of Care co-produced standards for in-patient care, summarised and adapted from NHS England^49^

	Core commitments				
1	Lived experience	Lived experience is integrated and valued at all levels.	7	Needs-led	Valuing peoples’ own understanding of their distress.
2	Safety	People feel safe and cared for on wards.	8	Choice	‘Nothing about me without me.’
3	Relationships	People have high-quality rights-based care with trusting relationships.	9	Environment	In-patient spaces reflect the value placed on people.
4	Staff support	Staff are supported to be present alongside people in their distress.	10	Things to do on the ward	Wide range of patient-request activities every day.
5	Equality	Inclusive care that values difference and promotes equity in access, treatment, and distress.	11	Therapeutic support	Offering a range of therapies and support that provide hope.
6	Avoiding harm	Actively seek to avoid harm and traumatisation.	12	Transparency	Honest and open conversations and naming difficult things.

Adapted from: https://www.england.nhs.uk/long-read/culture-of-care-standards-for-mental-health-inpatient-services/.

## Supporting information

Quinlivan et al. supplementary material Quinlivan et al. supplementary material

## Data Availability

The authors confirm that the data supporting the findings of this study are available within the article and/or its supplementary materials.

## Supplementary material

The supplementary material is available online at https://doi.org/10.1192/bjo.2025.10811

## Patient and public involvement

Research ideas for the grant application were developed together with our PPIE group (MS4MH-R). Our patient and carer advisory members (MS4MH-R) were involved throughout the research process. Our panel collaboratively developed the initial research questions, reviewed protocol and data extraction plans, results and contributed to interpretations, conclusions, implications and dissemination plans. People with lived experience in this area contributed to drafts, are co-authors and have completed a lived-experience commentary based on this review. There was PPIE input into our dissemination plan, which includes communicating key findings to relevant patient groups, carers and mental health services.
