# Umbrella review of psychosocial and ward-based interventions to reduce self-harm and suicide risks in in-patient mental health settings – ADDENDUM

**DOI:** 10.1192/bjo.2025.10907

**Published:** 2025-11-11

**Authors:** Leah Quinlivan, Jodie Westhead, Jane Graney, Fanyi Su, Sarah Steeg, Emma Nielsen, Eloise Curtis, Ellie Wildbore, Faraz Mughal, Rachel Elliott, Roger T. Webb, Nav Kapur

This article was originally published with a supplementary file omitted. The supplementary file has now been published along with this correction notice.

## Supporting information

Quinlivan et al. supplementary material Quinlivan et al. supplementary material
