# Supplementary material for: Umbrella review of psychosocial and ward-based interventions to reduce self-harm and suicide risks in in-patient mental health settings
Source: BJPsych Open. 2025 Sep 8;11(5):e196. doi: 10.1192/bjo.2025.10811 (PMC12451727; doi:10.1192/bjo.2025.10811)
Supplement: Quinlivan et al. supplementary material [file S2056472425108119sup001.docx]

**Title:** Moving beyond environmental adaptions for inpatient safety: Umbrella review of psychosocial and ward-based interventions to reduce self-harm and suicide risks in inpatient mental health settings: Lived Experience Commentary, Nielsen et al

**Authors:** Emma Nielsen,^1^ Sadika Asmal,^1^ Eloise Curtis,^1^ Dan Stears,^1^ Ellie Wildbore,^1^

1. National Institute for Health and Care Research (NIHR) Greater Manchester Patient Safety Research Collaboration, University of Manchester, UK

**Author statement:** EN, SA, EC, EW, have lived experience of receiving mental health inpatient care, self-harm and/or suicidal behaviour and are collaborators for the NCISH Culture of Care, Personalised Approach to Risk Programme and the National Institute for Health and Care Research, Greater Manchester Patient Safety Research Collaboration. DS is a lived experience collaborator with the NIHR GM PSRC (MS4MH-R), the Chair of the Greater Manchester Mental Health NHS Foundation Trust Patient Council. DS is also a patient representative for all mental health services, including inpatient care. EN is an academic self-harm and suicide prevention researcher and lived experience collaborator. They led the commentary and wrote the first draft. EC, EW, SA, DS contributed their views and experiences, and to subsequent drafts. All authors approved the final version.

**Funding**

This paper presents independent research funded by the National Institute for Health and Care Research (NIHR) Greater Manchester Patient Safety Research Collaboration (Grant Reference Number NIHR204295). The views expressed in this article are those of the authors and not necessarily those of NICE, NIHR, NHS, or the UK Department of Health and Social Care.

**Introduction**

The umbrella review seeks to synthesise systematic review evidence, to evaluate the effects of psychosocial interventions for self-harm and suicide prevention interventions in inpatient mental health settings. The review highlights a lack of robust, tailored interventions, as well as a stark disjunct between the research evidence and clinical practice. A number of questions remain outstanding, many of which arise from a lived experience perspective, which – as Quinlivan highlights – is rarely reported in the context of review work. Here, we present reflections, informed by this lens.

**Is intervention happening?**

Whilst the review seeks to evaluate the effects of interventions in inpatient settings, a question remains as to how much psychosocial work is currently happening within these contexts. In our experience, inpatient settings are often experienced as a missed opportunity, where meaningful intervention could have taken place but are often lacking. Similarly, we are mindful that there are instances in which inpatient hospitalisation can disrupt the delivery of outpatient or community-based interventions; community-based therapy might be halted, postponed, or cancelled on admission to hospital, even in instances where no inpatient psychosocial intervention is offered. This warrants exploration and understanding, not least as this disruption may be experienced as a destabilising un-planned ending or un-repaired rupture. There may be poor communication, or little to no ‘joined up thinking’ between inpatient and community teams and assumption can be made regarding the provision of intervention.

Where psychosocial interventions are offered, therapy often occupies just a single contact hour in a week. This leaves 167 hours within the inpatient week. Understanding these hours might be important to begin to unpack some of the conflicting evidence and increasing effectiveness of interventions; the wider culture of the ward is important. This includes ward-based approaches to understanding, managing, and responding to risk and whether ward-based responses are experienced as consistent with the work within psychology. For example, it can feel undermining if psychology is focused on learning to sit with feelings but ward-based responses to distress focus primarily around short-term sedating medication and distraction. Similarly, psychology might encourage a patient to use words to express distress, but if verbal communication and requests for support are not met by staff - but episodes of self-harm are responded to – this can be experienced as confusing and contradictory.

**Defining interventions – is it the same or something else?**

Within reviews, the trials grouped within the category of a given approach often vary considerably in their format and delivery. For example, ‘Dialectical Behavioural Therapy’ (DBT) in Nawaz et al., (1) included therapeutic interventions as short as 6 weeks (2), where standard DBT programmes are often delivered intensively over 12-months (e.g., 3), consisting of several therapeutic contacts across each week (4).

Some systematic reviews separate types of DBT (5), some do not (1). A question remains as to how far from ‘standard’ an intervention can be, while still being meaningfully referred to as intervention-based, intervention-informed or intervention-like. We know from experience that often when interventions are in place in inpatient settings they are presented in a group-based format, with sessions developed in-house, although based on established therapeutic manuals and principles. Given the scarcity of evidence identifying the active ingredient(s) within a therapy – should there be one – it is unclear how these formats could be considered evidence-based. While therapists within trials often receive sustained specialist training and supervised practice (3), it is unclear what training and support look like in informed-type examples in practice, and whether this experience would impact the effectiveness of treatments.

**‘Not one size fits all’**

It is often levelled that the question is not simply ‘what works?’ but ‘what works for whom, and when?’. Colloquially, we often hear the phrase ‘not one size fits all’, yet the criteria of the trials on which our evidence base is drawn are often narrow and restrictive (e.g., female; EUPD diagnosis). In our experience of ward-based interventions, psychology groups are often offered as a whole-ward approach, without any patient matching or conversations around individual needs, goals and personal suitability. It is unclear how these interventions might be experienced by those patients who fall outside of the typical participant profile of research trials or how effective the intervention may be. Our interventions might also require adaptation, translation, or revision to meet individual needs, for example to make interventions accessible, acceptable, and effective for neurodivergent people or those with learning support needs.

**What is success?**

Self-harm can be a persistent feature in someone’s life; someone may have an extensive history of self-harm at the point of hospitalisation. A patient might view their self-harm as a problem, but they might not. Self-harm might be an intervention target for the patient, but it might not be their priority, especially early on. Often patient views on trial outcomes – what constitutes ‘success’ – is not aligned to what is traditionally measured (6). Assessing effectiveness and efficacy will remain critically flawed until (co-produced) consensus is reached regarding both the most appropriate primary outcome(s) in research trials and the timeframe over which change might be seen.

Inpatient settings might offer an opportunity for stabilisation work or initial skills development. Considering the translation of evidence to practice, the planning and delivery of psychosocial interventions is often complicated in acute settings by uncertainty around the duration of admission. Indirect interventions might have considerable impact on self-harm. This may be downstream impact and would be difficult to capture within the trials and follow up periods we see; indirect intervention work might not include self-harm as an outcome of interest and therefore it could not fall within the review work.

How people engage with and respond to interventions is likely to be impacted by previous experience(s). It is important to remain mindful that people may have difficult histories with services and have experienced iatrogenic harm. It is difficult to consider how someone might trust a service that they had previously experienced as not being responsive, consistent, and safe.

**Building better together**

The umbrella review concluded that psychosocial interventions show promise for self-harm and suicide prevention but that there is a lack of robust evidence for interventions in mental health inpatient settings. Given this, we agree that meaningful co-design work for intervention planning is essential, alongside work to understand – from a patient perspective – the needs and priorities when thinking about self-harm intervention within the inpatient context. To be most effective this arguably needs to be broader in consideration than ‘just’ what happens within the therapy room, but also consider whole-ward culture approaches, ward-specific social factors and ‘triggers’, managing endings and transitions (including to community settings), and structural issues within and outside of healthcare (e.g., housing). Moving forwards, we argue that is important to explore in a more nuanced way what ‘recovery’ means to those who self-harm, what that looks like, which outcomes matter and what supports (i) not acting on self-harm thoughts, but also (ii) moving to a point where ego dystonic self-harming thoughts are no longer experienced.

**References**

1. Nawaz RF, Reen G, Bloodworth N, Maughan D, Vincent C. Interventions to reduce self-harm on in-patient wards: systematic review. BJPsych Open. 2021;7(e80):1–9.

2. Gibson J, Booth R, Davenport J, Keogh K, Owens T. Dialectical behaviour therapy-informed skills training for deliberate self-harm: A controlled trial with 3-month follow-up data. Behav Res Ther. 2014 Sep;60:8–14.

3. Bedics JD, Atkins DC, Comtois KA, Linehan MM. Treatment differences in the therapeutic relationship and introject during a 2-year randomized controlled trial of dialectical behavior therapy versus nonbehavioral psychotherapy experts for borderline personality disorder. J Consult Clin Psychol. 2012 Feb;80(1):66–77.

4. McDonell MG, Tarantino J, Dubose AP, Matestic P, Steinmetz K, Galbreath H, et al. A pilot evaluation of dialectical behavioural therapy in adolescent long-term inpatient care. Child Adolesc Ment Health. 2010;15(4):193–6.

5. Witt KG, Hetrick SE, Rajaram G, Hazell P, Taylor Salisbury TL, Townsend E, et al. Psychosocial interventions for self-harm in adults. Cochrane Database Syst Rev. 2021 Apr 22;4(4):CD013668.

6. Owens C, Fox F, Redwood S, Davies R, Foote L, Salisbury N, et al. Measuring outcomes in trials of interventions for people who self-harm: qualitative study of service users’ views. BJPsych Open. 2020 Feb 12;6(2):e22.
