# Supplementary material for: Umbrella review of psychosocial and ward-based interventions to reduce self-harm and suicide risks in in-patient mental health settings – ADDENDUM
Source: BJPsych Open. 2025 Nov 11;11(6):e276. doi: 10.1192/bjo.2025.10907 (PMC12641406; doi:10.1192/bjo.2025.10907)
Supplement: Quinlivan et al. supplementary material [file S2056472425109071sup001.docx]

**Supplementary material: Title:**

Moving beyond environmental adaptions for inpatient safety: Umbrella review of psychosocial and ward-based interventions to reduce self-harm and suicide risks in inpatient mental health settings

1. PRIOR Checklist

(Gates M, Gates A, Pieper D, et al. Reporting guideline for overviews of reviews of healthcare interventions: development of the PRIOR statement. *BMJ* 2022;378:e070849. doi:10.1136/bmj-2022-070849.)

| **Section**  Topic | **#** | **Item** | **Location reported** |
| --- | --- | --- | --- |
| **TITLE** | | | Pages |
| Title | 1 | Identify the report as an overview of reviews. | 1 |
| **ABSTRACT** | | |  |
| Abstract | 2 | Provide a comprehensive and accurate summary of the purpose, methods, and results of the overview of reviews. | 3 |
| **INTRODUCTION** | | |  |
| Rationale | 3 | Describe the rationale for conducting the overview of reviews in the context of existing knowledge. | 4 |
| Objectives | 4 | Provide an explicit statement of the objective(s) or question(s) addressed by the overview of reviews. | 4 |
| **METHODS** | | |  |
| Eligibility criteria | 5a | Specify the inclusion and exclusion criteria for the overview of reviews. If supplemental primary studies were included, this should be stated, with a rationale. | 5 |
|  | 5b | Specify the definition of ‘systematic review’ as used in the inclusion criteria for the overview of reviews. | 5 |
| Information sources | 6 | Specify all databases, registers, websites, organizations, reference lists, and other sources searched or consulted to identify systematic reviews and supplemental primary studies (if included).  Specify the date when each source was last searched or consulted. | 5-6 |
| Search strategy | 7 | Present the full search strategies for all databases, registers and websites, such that they could be reproduced. Describe any search filters and limits applied. | Supplentary materials |
| Selection process | 8a | Describe the methods used to decide whether a systematic review or supplemental primary study (if included) met the inclusion criteria of the overview of reviews. | 5 |
|  | 8b | Describe how overlap in the populations, interventions, comparators, and/or outcomes of systematic reviews was identified and managed during study selection. | 7 |
| Data collection process | 9a | Describe the methods used to collect data from reports. | 6 |
|  | 9b | If applicable, describe the methods used to identify and manage primary study overlap at the level  of the comparison and outcome during data collection. For each outcome, specify the method used to illustrate and/or quantify the degree of primary study overlap across systematic reviews. | 7 |
|  | 9c | If applicable, specify the methods used to manage discrepant data across systematic reviews during data collection. | 8 |
| Data items | 10 | List and define all variables and outcomes for which data were sought. Describe any assumptions made and/or measures taken to identify and clarify missing or unclear information. | Page 6 and supplementary material |
| Risk of bias assessment | 11a | Describe the methods used to *assess* risk of bias or methodological quality of the included systematic reviews. | 7 |
|  | 11b | Describe the methods used to *collect* data on (from the systematic reviews) and/or *assess* the risk of bias of the primary studies included in the systematic reviews. Provide a justification for instances where flawed, incomplete, or missing assessments are identified but not re-assessed. | 7 |
|  | 11c | Describe the methods used to *assess* the risk of bias of supplemental primary studies (if included). | NA |
| Synthesis methods | 12a | Describe the methods used to summarize or synthesize results and provide a rationale for the choice(s). | 8 |
|  | 12b | Describe any methods used to explore possible causes of heterogeneity among results. | 8 |
|  | 12c | Describe any sensitivity analyses conducted to assess the robustness of the synthesized results. | NA |
| Reporting bias assessment | 13 | Describe the methods used to *collect* data on (from the systematic reviews) and/or *assess* the risk of bias due to missing results in a summary or synthesis (arising from reporting biases at the levels of the systematic reviews, primary studies, and supplemental primary studies, if included). | 7 |
| Certainty assessment | 14 | Describe the methods used to *collect* data on (from the systematic reviews) and/or *assess* certainty (or confidence) in the body of evidence for an outcome. | 6-7 |
| **RESULTS** | | |  |
| Systematic review and supplemental primary study selection | 15a | Describe the results of the search and selection process, including the number of records screened, assessed for eligibility, and included in the overview of reviews, ideally with a flow diagram. | 9 |
|  | 15b | Provide a list of studies that might appear to meet the inclusion criteria, but were excluded, with the main reason for exclusion. | Supplementary materials and page 9 |

1. **Supplementary material: Search strategy terms and approach**

We adapted from the NICE guidelines^1^, and Cochrane reviews of psychosocial assessments for self-harm.^1-2^ We used an intentionally broad initial approach to increase the likelihood of capturing a wide range of self-harm and suicide prevention reviews. Search terms were adapted for each database together with a specialist librarian and under supervision of senior authors (RW, NK). Selection of reviews was narrowed though the application of our PICO inclusion criteria (Table 1 in text).

| Table 1. Search terms adapted for databases from January 2013 to October 19, 2023 | |
| --- | --- |
| Database | Search terms |
| CINAHL | 1.(Suicidal Behavio*) OR Suicide OR (Suicidal Idea*) OR (Attempted Suicide) OR (Self Poisoning) OR Suicidology OR (suicide risk) OR parasuicide OR (suicide prevent*) OR (reduce suicid*) OR (self harm*) OR selfharm*.ti  OR  2.(self injur*) OR selfinjur* OR (self mutilat*) OR selfmutilat* OR suicid* OR (self destruct*) OR selfdestruct* OR (self poison*) OR selfpoison* OR (self adj cut*) OR overdose* OR (self immolat*) OR (self immolat*) OR selfinflict* OR (self inflict*) OR (auto mutilat*) OR automutilat*.ti  OR  3.((self N2 (cut OR cuts OR cutting OR cutter OR burn OR burns OR burning OR bite OR bites OR biting OR hit OR hits OR hitting)) OR head bang* OR headbang* OR (NSSI OR nonsuicid* OR non suicid* N2 (self* OR injur*)).ti  AND  4.Review.ti |
| EMBASE | 1.“Suicidal Behavio*” OR Suicide OR “Suicidal Idea*” OR “Attempted Suicide” OR “Self Poisoning” OR Suicidology OR “suicide risk” OR parasuicide OR “suicide prevent*” OR “reduce suicid*” OR “self harm*” OR selfharm* OR “self-harm”:ti.  OR  2.OR “self injur*” OR selfinjur* OR “self mutilat*” OR selfmutilat* OR suicid* OR “self destruct*” OR selfdestruct* OR “self poison*” OR selfpoison* OR “self NEXT/2 cut*” OR overdose* OR “self immolat*” OR OR selfinflict* OR “self inflict*” OR “auto mutilat*” OR automutilat*:ti.  OR  3.((self NEXT/2 (cut OR cuts OR cutting OR cutter OR burn OR burns OR burning OR bite OR bites OR biting OR hit OR hits OR hitting)) OR head bang* OR headbang* OR (NSSI OR nonsuicid* OR non suicid*) NEXT/2 (self* or injur*)):ti.  AND  4.Review:ti. |
| PSYCINFO | 1. (Suicidal Behavio* or Suicide or Suicid* or Suicidal Idea* or Attempted Suicide or Self Poisoning or Suicidology or suicide risk or parasuicide* or suicide prevent* or reduce suicid* or self harm* or selfharm* or self-harm*).m_titl. 2. ((self injur* or selfinjur* or self mutilat* or selfmutilat* or self destruct* or selfdestruct* or self poison* or selfpoison* or self) adj 2 cut*) or overdose* or self immolat* or selfinflict* or self inflict* or auto mutilat* or automutilat*).m_titl. 3. ((self adj2 (cut or cuts or cutting or cutter? or burn or burns or burning or bite or bites or biting or hit or hits or hitting)) or head bang* or headbang* or (NSSI or ((nonsuicid* or non suicid*) adj2 (self* or injur*)))).m_titl. 4. 1 or 2 or 3 5. review.m_titl. 6. 4 and 5 7. limit 6 to yr="2013 - 2023" |
| MEDLINE | 1. Suicidal Behavio* OR Suicide OR Suicidal Idea* OR Attempted Suicide OR Self Poisoning OR Suicidology OR suicide risk OR parasuicide OR suicide prevent* OR reduce suicid* OR self harm* OR selfharm*.ti   OR   1. self injur* OR selfinjur* OR self mutilat* OR selfmutilat* OR suicid* OR self destruct* OR selfdestruct* OR self poison* OR selfpoison* OR self NEAR/2 cut* OR overdose* OR self immolat* OR self immolat* OR selfinflict* OR self inflict* OR auto mutilat* OR automutilat*.ti   OR   1. self NEAR/2 (cut OR cuts OR cutting OR cutter OR burn OR burns OR burning OR bite OR bites OR biting OR hit OR hits OR hitting)) OR head bang* OR headbang* OR (NSSI OR nonsuicid* OR non suicid* NEAR/2 (self* OR injur*)).ti   AND   1. Review.ti |
| CDSR | Record Title: Suicidal NEXT Behavio* or Suicide or Suicid* or Suicidal NEXT Idea* or Attempted NEXT Suicide or Self NEXT Poisoning or Suicidology or suicide NEXT risk or parasuicide* or suicide NEXT prevent* or reduce NEXT suicid* or (self harm*) or( selfharm*) or (self-harm*)OR Record title: (self injur*) or selfinjur* or (self mutilat*) or selfmutilat* or (self destruct*) or selfdestruct* or (self poison*) or selfpoison* or self adj 2 (cut* or overdose*) or (self immolat*) or selfinflict* or (self inflict*) or (auto mutilat*) or automutilat* OR Record title: ((self adj2 (cut or cuts or cutting or cutter? or burn or burns or burning or bite or bites or biting or hit or hits or hitting)) or head bang* or headbang* or (NSSI or ((nonsuicid* or non suicid*) adj2 (self* or injur*)))) AND Record title: Review. |

**References**

1. National Institute for Clinical Excellence. Self-harm: assessment, management, and preventing recurrence. NICE Guidelines, NG225. 2022.
2. Witt KG, Hetrick SE, Rajaram G, Hazell P, Salisbury TLT, Townsend E, et al. Psychosocial interventions for self-harm in adults—Witt, KG - 2021 | Cochrane Library. 2021. Available from: <https://www.cochranelibrary.com/cdsr/doi/10.1002/14651858.CD013668.pub2/full>

| Table 2. Inpatient settings: quantitative study characteristics and additional results | | | | | | | | |  |  |
| --- | --- | --- | --- | --- | --- | --- | --- | --- | --- | --- |
| Ref | **Interventions as listed** | **Control** | **Aprox. Follow-up** | **Aprox. intervention length summary** | **Outcomes** | **Designs** | **Total studies** | **Results** | **Heterogeneity** | **Risk of bias** |
| Fox2020 | Interventions for suicide and self-injury (e.g., DBT/CBT, psychotherapy, safety planning) | Active control, placebo, no-treatment | *Mdn*= 12 weeks | 12 weeks | Suicide, suicide attempts, NSSI, self-harm | RCT | 591 | **Overall analyses [interventions combined] Suicide attempts**: Treatments did not significantly reduce suicide attempts (0.90 [0.87,1.11]) on binary or continuous outcomes (*g* =-0.10 [-0.27, 0.06]).**Self-harm**: Treatment did not significantly reduce self-harm (RR=0.93 [0.84, 1.04] binary; or on continuous outcomes [g=-0.15[-0.47,0.10])  **Suicide**: Interventions did not significantly reduce suicide (RR=0.89 [0.76, 1.04])  **NSSI**: Interventions did not significantly reduce NSSI using a discrete: (RR = 1.11 [0.98, 1.27]), or continuous outcome: (*g* = -0.07 [-0.18, 0.04]).DBT significantly reduced severity/intensity of combined SITB, and self-harm, and slightly reduced SITB-related hospitalisations. DBT did not reduce suicidal ideation, attempts, or NSSI. No studies on death. CBT/CBT reduced combined outcome (continuous version), and did not reduce suicide attempts or death. Too few studies to look at other outcomes. Checking-in programmes: reduction in suicidal ideation and attempts, but small numbers. Pooling found worse outcomes overall for checking-in programmes. Analysed treatments (medication, CT/CBT, DBT, psychotherapy, checking-in, psychoanalysis/insight-based therapy), problem solving therapy, safety planning/means safety, inpatient hospitalisation) and outcomes together and separately. All age groups, treatment components in moderator analyses. | Random effects meta-analysis; Suicide attempts: Low heterogeneity and minimal publication bias Suicide death: Between-study heterogeneity and publication bias were low. NSSI: Between-study heterogeneity and publication bias were not detected. Self-harm: Results indicated low heterogeneity and minimal publication bias, but moderate for continuous outcomes. Combined outcomes: Little between-study heterogeneity and publication bias were detected. | CROB: low for publication bias, More than half of the effect sizes (57.58%) were associated with weak study quality. More than a quarter of the effect sizes (36.21%) were from studies with moderate quality, with only 6.22% of the effect sizes from studies with strong quality. |
| DeCou2019 | Dialectical behaviour therapy for treatment of suicidal behaviour | TAU, waitlist, active control | Two weeks to 12 months | 13 weeks to 12 months | Suicidal behaviour, NSSI, self-harm [composite used ‘self-directed violence’ ] | RCT | 18 | Self-directed violence: DBT significantly reduced self-directed violence compared to control conditions: (Weighted Mean Effect Size, d = -.324, 95% CI = -.471 to -.176). | Random-effects meta-analysis. Between-study heterogeneity was low among studies included in meta-analyses of suicidal and NSSI behaviours (*I*^2^ = 0.00%). | No evaluation of bias. Mix of inpatient and outpatient settings, populations, and age. |
| Hawton2016 | Psychosocial interventions following self-harm: CBT based psychotherapy (CBT and problem solving therapy);  Dialectical behaviour therapy; case management, brief interventions. | TAU, lower intensity active control | Six ,12, to 24 months follow-up | Insufficient information | Self-harm and suicide | RCT | 29 | Self-harm: significant treatment effects for CBT based psychotherapy associated for fewer repeat self-harm episodes at 6 months’ (odds ratio 0·54, 95% CI 0·34–0·85) and at 12 months’ follow-up (0·80, 0·65–0·98). No significant reduction in the frequency of self-harm, or suicide. No significant treatment effects for DBT on repeat self-harm compared to a control group at 6 or 12 months. Significant treatment effects for DBT in the reducing the frequency of self-harm (mean difference –18·82, 95% CI –36·68 to –0·95). No treatment effects for reducing suicide*. Case management and postcards did not reduce self-harm repetition or suicide.  *few suicide deaths recorded. | Heterogeneity:  CBT 3 months and 12 months: repeat self-harm:  *I*^2^=36%; 0%;  Suicide: i2=0%;  DBT: 6months, 12 months,  *I*^2^=59%, 66%  Suicide: p=0.51;  Case management repeat self-harm:  *I*^2^=54%, and 0% for suicide; Postcards; repeat self-harm post intervention and 12 months:  *I*^2^=51%, 49%; suicide post intervention: *I*^2^=12%.  . | CROB: publication bias; performance bias. Quality of evidence was moderate to very low, biases typically related to blinding, which is challenging in psychosocial interventions.  Settings/population: Adults, cross-settings (mix of hospital presentations, admissions to ED, medical unit, or acute psychiatric ward; community referrals, PD service, addiction and mental health unit, DBT service; CBT (hospital admissions, ED presentations, or acute psych unit); DBT: PD service, addiction and mental health unit, specialist DBT; Case management: presentations to hospital, admissions to hospital or clinical services, presentations to ED, acute ED units, intensive care or psychiatric emergency units.  Postcards: admission to psychiatric based toxicology service, psychiatric emergency services, ED.  Most of this evidence comes from hospital emergency department or outpatient settings |
| Witt2021 | Psychosocial interventions for self-harm (e.g., CBT-based interventions, DBT, Mentalisation-based therapy, emotion-regulation, and others) See Witt et al. (2021) | TAU, or other comparators (no treatment, enhanced usual care) | CBT: brief *Mdn*= 3 months. | 0, 6, and 12 months after the treatment finished. | Repeated self-harm, suicide | RCT | Meta-analysis of 44 studies of psychosocial interventions for adults. | **CBT-based psychotherapy vs TAU: Repeat self-harm:** CBT-based psychotherapy may reduce repeat self-harm post-intervention (OR 0.35, 95% CI 0.12 to 1.02; participants= 238; studies = 4;  *I*^2^ = 0%). Six-month follow-up: 12 trials, CBT-based psychotherapy may reduce repeat self-harm (OR 0.52, 95% CI 0.38 to 0.70; N = 1260; k = 12; I2 =2%). 12 months (9 trials): CBT-based psychotherapy may reduce repeat self-harm (OR 0.81, 95% CI 0.66 to 0.99; N = 2458; k = 9;  *I*^2^ = 0%; Analysis 1.3). **Frequency of self-harm:** No effect found for CBT-based psychotherapy on frequency of repeat self-harm by the post-intervention assessment (4 studies). There was evidence of an effect for CBT-based psychotherapy by the six-month assessment (MD -0.71, 95% CI -1.32 to -0.11; N =118; k = 4;  *I*^2^ = 0%) .There was evidence of an effect for CBT-based psychotherapy by the 12-month assessment in a single trial (mean 1.18, SD 4.22, n = 40 versus mean 4.58; SD 8.37; n = 33; MD -3.40, 95% CI -6.54 to -0.26; N = 73; k = 1;  *I*^2^ = not applicable. **Suicide:** No effect on suicide by final follow-up up to 24 months (16 trials).  **Group-based cognitive behavioural therapy (CBT)-based psychotherapy versus TAU or another comparator.** No evidence to suggest that group-based CBT-based psychotherapy reduces repetition by the post intervention assessment, or by six-month, or 12-month follow-up, for repeat self-harm or suicide.  **Dialectical behavioural therapy (DBT) versus TAU or another comparator**  No evidence of an effect for standard DBT  compared to either TAU or other psychotherapy for reducing repeat self-harm by the post-intervention assessment (very low certainty evidence). No evidence of an effect for DBT on 12 month-follow-up compared to comparator. DBT vs TAU or alternative therapy: associated with reduced frequency of repeat self-harm by the post-intervention assessment (MD -5.00, 95% CI -8.92 to -1.08; N = 659; k = 7; *I*^2^= 49%). However, there was no effect found by the six-month follow-up assessment in one trial.  No evidence of an effect for DBT on suicide deaths at either post- Intervention,12-month, or 24- month follow-up period, compared to TAU. **DBT group-based skills training**  No evidence of an effect for DBT group-based skills training only compared with standard DBT on suicide reattempts or non-suicidal self-injury by the 12-month assessment. No evidence for an effect on the frequency of suicide re-attempts or episodes of NSSI by post-intervention, nor 12 month follow-up (1 trial). **DBT individual therapy vs standard DBT: No** evidence of an effect for DBT individual therapy compared with standard DBT on suicide reattempts.  **Mentalisation-based therapy (MBT) versus TAU or another comparator** MBT may reduce repeat self-harm by the 18-month treatment period (data from author correspondence) (18/71 versus 31/63; OR 0.35, 95% CI 0.17 to 0.73; N = 134; k = 1;  *I*^2^ = not applicable). Evidence rated as high certainty. Evidence of an effect for MBT on reducing the frequency of self-harm the post-intervention assessment (author correspondence data) according to data (mean 0.38, SD 0.38, n = 71 versus mean 1.66, SD 2.87, n = 63; MD -1.28, 95% CI -2.01 to -0.55; N = 134; k = 1;  *I*^2^ = not applicable). **Emotion-regulation psychotherapy versus TAU or another comparator** Some evidence to suggest that group-based emotion-regulation may reduce repeat self-harm by post-intervention assessment (2 trials), rated as moderate certainty. (OR 0.34, 95% CI 0.13 to 0.88; N = 83; k =2;  *I*^2^ = 0%). Grade: moderate certainty.  No evidence of an effect on frequency of repeat self-harm by the post-intervention assessment. **Psychodynamic psychotherapy versus TAU or another comparator:** No evidence of an effect for psychodynamic psychotherapy by the post-intervention in one trial (Grade: moderate certainty). **Case management versus TAU or another comparator** There was no evidence of an effect for case management on repeat self-harm (5 trials), or suicide by the post intervention assessment or 12-month follow-up. **Structured general practitioner (GP) follow-up versus TAU or another comparator.** No evidence of an effect for structured GP follow-up on repeat self-harm by post-intervention assessment (low certainty evidence). **Brief emergency department-based** **interventions versus TAU or another comparator** No evidence of an effect for brief CAMS and DBT-based interventions on repeat self-harm by the 12-month follow-up (1 trial), or frequency of repetition (1 trial). **Brief guided Integrated Motivational-** **Volitional-focused intervention**  No evidence of an effect on repeat self-harm by 6-month follow-up (1 trial), or frequency of repeat self-harm (1 trial), or on time (1 trial), or deaths by suicide by the 6-month follow-up (1 trial).  **Remote contact interventions versus TAU or another comparator** No evidence of an effect for emergency cards on repeat self-harm by post intervention assessment (2 trials rated as low certainty evidence), or by the 12-month follow-up (1 trial), or frequency of repeat self-harm, or time of repetition, or suicide (1 trial). **Coping cards** No evidence for an effect of coping cards on the proportion of suicide re-attempts by the post-intervention time (Grade: moderate certainty). **GP letters:** no evidence for an effect for GPs letters for reducing repeat self-harm by 12 months or on time to repeat (1 trial). **Postcards:** no evidence of an effect for postcards on repeat self-harm by post-intervention (Grade: very low quality certainty of evidence),or by the 12 month assessment (2 trials), or the frequency of repeated self-harm (3 trials), or suicide by post-intervention assessment (4 trials), or by the 12 month assessment (1 trial). **Telephone contact**  No evidence for an effect of telephone contact on repeat self-harm by the post-intervention assessment (1 trial) (Grade: low certainty evidence), or on repeat self-harm by 12 or 24 months, or suicide by the post-intervention or 24-month assessment. **Telephone contact combined with emergency cards and letters:** No evidence for an effect for telephone contact combined with emergency cards on repeat self-harm by post-intervention assessment (1 trial, of moderate certainty), or frequency of repeated self-harm. **Telephone-based psychotherapy:** No evidence for an effect on repeat self-harm by post-intervention assessment (2 trials, rated as low certainty of evidence), or on repetition by 6 months, or 12 months (1 trial). **Provision of information and support versus TAU or another comparator:** No evidence for an effect for information and support on repeat self-harm by 18 months, post intervention assessment (2 trials, rated as very low certainty evidence). **Other multimodal interventions versus TAU or another comparator:** No evidence for an effect on a package of interventions on repeat self-harm at post intervention, rated as very low certainty evidence). Some evidence of an effect for reducing the frequency of repeat self-harm at post intervention (1 trial) (mean 0.08, SD 0.28, n = 60 versus mean 0.82, SD 1.89, n = 50; MD -0.74, 95% CI -1.27 to -0.21; N = 110; k = 1; I *I*^2^ = not applicable; Gysin-Maillart 2016). No effect for on deaths by suicides by the post-intervention assessment in any trial. **Continuity of care by the same therapist:** No evidence of an effect on repeat self-harm by 12 months (1 trial), or suicide (1 trial). **Interpersonal problem-solving therapy:**  No evidence for an effect on reducing repeat self-poisoning by 12 months (1 trial). **Behaviour therapy:**  No evidence of an effect on repeat self-harm by 24 month follow-up. **Intensive in- and outpatient treatment:** No evidence of an effect for intensive in-and-outpatient treatment for reducing repeat self-harm by 12-month follow-up, or on the frequency of repetition, or time to self-harm, or suicide by 12 months. **General hospital admission:** No evidence for an effect of hospital admission on repeat self-harm at the post-intervention assessment (Grade moderate certainty), or at the 4-month follow-up (1 trial). I**ntensive outpatient treatment:** No evidence for an effect of intensive outpatient treatment on repeat self-harm by four- or 24-month follow-up. **Home-based psychotherapy and telephone contact:**  No evidence for an effect on repeat self-harm by 12 months follow-up.  **Long-term therapy:**  No evidence of an effect on repeat self-harm (1 trial) | Investigation of heterogeneity when over i *I*^2^=75%, reported in previous box. | CROB:  Most trials rated as having some or high risk of bias (84.2%)  CBT: risk of bias high for one trial and some bias for other trials. Evidence rated as low certainty according to GRADE criteria.  Six trials included inpatient settings, 4/6 received treatment and outpatient follow-up, 2 studies received treatment during inpatient stays. |
| Hou2022* | Social support interventions to prevent suicide (postal, text, face-to-face, email) | TAU, waitlist | Six months to 12.8 years | Three months to 5 years | Suicide, suicide attempts | RCT | 14 suicide attempts  10 for suicide | **Suicide:** Significant treatment effects towards reduction in suicide deaths (overall pooled RR was 0.48 (95% CI 0.27 to 0.85, p=0.01). [52% reduction in intervention group compared to TAU for suicide]. Subgroup analyses: format: Face to face interventions significantly reduced the risk of suicide with a pooled RR of 0.16 (95% CI 0.05. to 0.53), different methods did not. Population: Interventions targeting people who had attempted suicide reduced their risk of suicide (RR=0.24, 95% CI 0.10 to 0.58). Interventions targeting other participants did not reduce suicide significantly. Moderate but non-significant heterogeneity (suicide). Suicide attempts: No significant treatment effects for attempted suicide and significant heterogeneity (I^2^=52%, p=0.01). | Random-effects meta-analysis; heterogeneity: Suicide, intervention vs control:  moderate but non-significant ( *I*^2^=17%, p=0.30). Suicide attempts: Significant amount of heterogeneity between the studies (*I*^2^=52%, p=0.01). | CROB: Suicide attempts: Three studies: high quality; Suicide: five were of high quality; Main risks were detection bias and attrition bias.  Suicide data settings: inpatient facilities (Luxton 2019, Motto 1977, Motto & Bostrom, 2001-patients discharged from inpatients), Military installations, hospitals, emergency departments, crisis centre, primary care. 1/10 populations were adolescents. |
| Sobanski2021 | Psychotherapeutic interventions to prevent suicide re-attempts (e.g., CBT, DBT, interpersonal psychotherapy, problem solving) | TAU | Zero to 24 months | 1 hour to 5 years | Suicide attempts and suicides composite | RCT | 18 | Suicide attempts and suicide (composite): Significant difference in number of suicide re-attempts between psychotherapeutic interventions, and TAU or other control conditions (RR = 0.66; 95% CI 0.48–0.90; Z = 2.63, p = 0.008; OR 0.56, CI 0.36–0.84; p = 0.006. Suicide attempts: CBT vs TAU (19 studies):  significant difference PT and TAU, RR = 0.66; 95% CI 0.48–0.90; Z = 2.61, p = 0.009; OR 0.53, CI 0.34–0.83; p = 0.005. The between-study heterogeneity was low (I2 = 28%). Psychodynamic interventions  (MBT, brief psychodynamic interpersonal therapy) vs control: significant impact on the number of suicide re-attempts, RR = 0.21; 95% CI 0.08–0.57; Z = 3.08, p = 0.002; OR 0.17, CI 0.06–0.45; p = 0.0004. The between-study heterogeneity was low ( *I*^2^ = 30%).Better results seen for longer follow-up for CBT. No significant reductions in suicide attempts with DBT or solely problem-solving strategies.  Heterogeneity was moderate (*I*^2^ = 51%). | Random-effects meta-analysis: Heterogeneity: Pooled psychotherapy: Suicide re-attempts: The between-study heterogeneity was moderate ( *I*^2^= 51%).CBT Vs TAU suicide re-attempts: The between-study heterogeneity was low (I2 = 28%).Psychodynamic interventions vs controls: the between-study heterogeneity was low (*I*^2^ = 30%). | CROB: Potential for publication bias and other biases across studies (e.g., blinding, selection bias, detection bias, and other bias). Heterogenous samples (psychiatric diagnosis, history of suicidal behaviour, psychiatric medications, populations, demographic characteristics, intervention and follow-up length, training and experience of practitioners  Mixed settings and inadequately reported. outpatient inpatient and outpatient, community, ED. Only Davidson 2006, 2010 (inpatient and outpatient), Ghahramanlou-Holloway 2020; La Croix 2018, mentioned inpatient settings. |
| Yiu2021 | Psychosocial interventions to reduce risk of suicide and self-harm in psychiatric inpatients (CBT, DBT, diary entries, Insight-orientated therapy, cognitive restructuring, peer support for suicide prevention, creating coping skills training). | TAU, Enhanced usual care, or active treatment | 2 weeks to 2 years | 4 days to 6 months | Suicidality (main outcome), self-harm (no studies had self-harm as an outcome)  Suicide, suicide attempts | RCT | 10 | Suicide attempt (secondary outcome): No significant difference between treatment conditions in the meta-analysis for suicide attempts at three-to-six-month follow-up (RR= 0.92, 95%CI=0.41 to 2.06, *Z* = 0.18, *p*=0.86, *I*^2^= 0%). No studies using the outcome of self-harm. No significant difference between treatment conditions in reducing suicide both at post-therapy (SMD =–0.14, 95% CI =– 0.38 to 0.10, Z =1.12, p=.26) and at follow-up (SMD=0.22, 95% CI =– 0.15 to 0.59, Z =1.18, p=.24). Heterogeneity was low ( *I*^2^ =10% and 0% respectively) for both analyses. | Random-effects meta-analysis  Suicide: pre-post and vs controls: Heterogeneity was low ( *I*^2^ =10% and 0% respectively) for both analyses. Suicide attempts: 0% heterogeneity | CROB: Variable risk of bias, all studies: high risk of bias for blinding, several studies had high risk of bias for missing outcome data and selective reporting. |

Abbreviations: CT= controlled trials; CCT = controlled cohort studies; CBT= cognitive behaviour therapy; CAT; Cognitive analytic therapy; CPDpd= Cognitive behaviour therapy adapted for people who have received a personality disorder diagnosis. CROC: Cochrane Risk of Bias tool; EUC= Enhanced usual care; MBT; Mentalisation based therapy; DBT= dialectical behaviour therapy; RCT=Randomised controlled trials; ITS= Interrupted times series designs; Obs.= observational study design; PP= Pre-post, QED= Quasi-experimental designs; TAU = treatment as usual; CROB: Cochrane Risk of Bias.

| Table 3. Inpatient settings: Study characteristics and additional narrative results | | | | | | | |  |  |
| --- | --- | --- | --- | --- | --- | --- | --- | --- | --- |
| Short reference | **Intervention as listed in the paper.** | **Control** | **Aprox. Follow-up** | **Aprox. intervention length** | **Outcomes** | **Designs** | **Results** | **Heterogeneity** | **Bias** |
| Luxton2013 | Post discharge follow-up contacts to prevent suicide and suicidal behaviour.  Post-discharge suicide prevention interventions that involve follow-up (postal, phone calls, in-person visits, electronic, planned visits). | No follow-up | 1 month to 5 years | 1 month to 5 years | Suicide, suicide attempts, self-harm | RCT, Quasi-experimental  8 original studies, 2 follow-up analyses, 1 secondary analysis. | Based on three out of 11 studies, the authors suggest that repeated follow-up contacts may reduce suicidal behaviours. However, the authors found that four studies, had mixed or nonconclusive results, and two did not show preventative effects. Wide range in timing, number of and frequency of contacts (e.g., one to 24 follow-up contacts, and time duration ranged from 1 month to 5 years.  Follow-up contacts may reduce suicidal behaviour, but some inconclusive or mixed results across studies. Two did not show any effect and there was a wide range in timing, frequency of contact and follow-up. Indicates potential for brief follow-up. 3/11 studies showed a reduction in repeated suicide attempts, and two prevented deaths by suicide. Four studies were inconclusive and 2 did not show preventative effects. | Not discussed  Notes: mixed settings, emergency department and inpatients. Mixed outcomes. | Not discussed.  Most of the evidence comes from studies with patients discharged from emergency care. |
| Donker2013 | Suicide prevention in schizophrenia spectrum disorders and psychosis  Psychosocial interventions for reducing suicidal behaviour (psychoeducation, psychotherapy, case management, counselling, or community treatment) [schizophrenia spectrum/psychotic disorders]. | TAU |  | 1 session to 24 months | Self-harm, suicide, suicide attempts and suicidal behaviour | 10 papers and 11 Controlled trials | **Self-harm:** No significant differences in self-harm between Integrated motivational interviewing plus CBT intervention and control group. Suicide attempts: no significant differences between interventions (integrated treatment and TAU; CBT+TAU, supportive counselling +TAU) and TAU. Suicide: no significant differences in deaths by suicide and controls.  **Suicide attempts**: No significant difference between interventions and controls for psychosocial interventions (3 studies); Self-harm: no significant difference in self-harm between control and intervention group (1 study) (population: schizophrenia spectrum disorder and dependence on drug or alcohol misuse); **Suicide:** no significant differences in deaths by suicide between intervention and controls (8 studies). | Discussed for samples, screening, study population, outcome measures. | Jadad’s quality criteria (Jadad et a. (1996). Most studies measured as adequate. Variable reporting. Mixed settings: in-patient and out-patient settings, age from 15 to 65), all with schizophrenia spectrum disorders and psychosis. |
| Nawaz2021 | Interventions to reduce self-harm on in-patient wards.  (DBT, Problem solving therapy, Skills training, unified protocol, phone based positive psychology, post-admission cognitive therapy, ward-based interventions, mixed interventions) | Not reported | Three days to 6 months | 3 days to 6 months | self-harm | Pre-post designs (majority), RCT, CT, ITS  23 studies, 15/23= therapeutic interventions; 6/23=ward environment; 2/23= combination of ward and therapeutic techniques. | DBT was the most frequently implemented and effective therapeutic intervention (7 out of 8 studies). Three ward based-interventions reduced self-harm. Two studies that combined therapeutic and ward-based approaches reduced self-harm. Primary study quality varied, interventions poorly reported and overall evidence was weak. 17/23 rated as weak studies, 5/23 as moderate, and 1/23 as strong quality (RCT, pre-post designs)  **Interventions:**  DBT adapted for in-patient settings: 7/8 studies had significant reductions in self-harm;  Skills to enhance positivity (STEPs) and Systems training for Emotional Predictability and Problem Solving (STEPPS) therapy: 2 studies led to significant reductions in hospital admissions for self-harm (pre-post designs)  Problem solving therapy  2 (RCTS), 1 with self-harm as an outcome, no impact on rates of self-harm;  Other: post-admission CT, unified protocol, phone-based positive psychology: no impact on self-harm.  **Ward-based**  Safewards intervention: Pre-post designs (2 studies): significant reduction in suicide and self-harm; CT: no significant reduction in self-harm.  Staff training: 2/3 studies focusing on staff training significantly reduced self-harm incidents. 1/3 did not report findings. Additional nurses on acute wards assisting with implementation of changes according to a model of conflict and containment for 1 year; Collaborative problem-solving training for nurses: significant decrease in self-harm incidents (retrospective study of staff surveys and hospital records). | Discussion of heterogeneity of study design, interventions, and outcome measures as justification for narrative analysis. | Effective Public Health Practice Project quality assessment tool. One/23 intervention papers had strong quality rating, 5 had a moderate quality rating, and 17 rated as weak due to selection bias and masking. Only one study had a strong design, other studies used simple pre-post designs, with small sample sizes, several without controls.  Settings: all inpatient settings, no restrictions on age, psychiatric condition. Mixed interventions: one of two studies combined a therapeutic approach and ward based changes (changing how nurses were placed on wards, introducing recreational, boundaries for certain ward areas, and therapeutic activities): significant reduction in self-harm; second intervention (regular twilight shift, structured evening activity) reduced self-harm. |
| Mann2021 | ‘Improving suicide prevention through evidence-based strategies’  Brief suicide prevention interventions (brief contact interventions, care coordination, safety planning, and other brief therapies) | TAU | 8 months to 24 months | 2 years to 10 years | suicide, suicide attempts | 97 RCT and 30 epidemiologic studies (observational quasi experimental time-series with controls). | Overviews one system-wide healthcare change (before-after study), implementing evidence-based recommendations reduced suicide rates (UK). US study: staff education and screening of suicide risk in all psychiatric patients: reduced suicide rates. Treatments: CBT: decreased suicide risk and suicide attempts; DBT prevented suicide attempts, and ‘lessoned medical consequences of self-harm’; psychodynamic psychotherapies prevent suicidal or self-harm behaviours. Group psychotherapies: reduced suicide in one study. Contact/outreach following discharge: Postcards: prevented suicide attempts (2/4 studies); Enhanced treatment engagement and compliance via follow-up calls reduced suicide attempts (4/5 studies); Caring text messages to military personnel: lowered suicide attempts; Safety plans: ED presentations with follow-up: 45% reduction in suicidal behaviours vs TAU. |  | Heterogeneity discussed in the discussion for psychiatric illness, proportion of males, sample sizes, outcome measures, and complex interventions.  Studies included mixed-settings and interventions. Relevant to inpatient settings: pre-discharge education and follow-up contact and outreach following suicidal crises; specific psychotherapies (CBT, DBT) reduced suicidal behaviour.  Primary studies: wide variability in quality and quantity of data available, wide heterogeneity of study populations for psychiatric illness, proportion of high-risk patients (history of suicide attempt, age, ethnicity, men). Lack of information on indigenous populations, older people, and psychiatric disorders, small samples, lack of replication; difficult to determine effective components due to complex interventions with multiple components. |
| Wand2022 | Evidence-based aftercare for older adults following self-harm  Social support interventions to prevent suicide (postal, text, face-to-face, email) | TAU without follow-up or care additional components | 24 months | Insufficient information | Suicide, suicide attempts | Historical observational cohort | 20 studies, 2/20 study evaluated the ‘Elderly Suicide Prevention Programme’ (Hong Kong): comprehensive assertive aftercare programme, with urgent referral of case manager and psychogeriatric appointment for older adults, using a historical control: results indicated a 52% reduction in intervention group compared to TAU for suicide. | Heterogeneity discussed in terms of study design, interventions, and outcomes. | Quantitative research: Alberta Heritage Foundation for Medical Research Standard Quality Assessment Criteria (Kmet et al., 2004).  In the total review, only six studies rated as high-quality, but only one poor quality study was relevant for this paper. Setting: hospital to community psychiatric care. Strength of evidence: poor, single study with significant methodological limitations, small absolute risks reduction which may be due to Type 1 error. Limited evidence-base for aftercare in older people. |
| Rozek2022 | Addressing co-occurring suicidal thoughts and behaviours and posttraumatic stress disorder in evidence-based psychotherapies for adults  Psychotherapeutic interventions to reduce suicide re-attempts (e.g., CBT, DBT, interpersonal psychotherapy, problem solving) | TAU, waitlist, enhanced care | Insufficient reporting | Insufficient information | Suicidal behaviours | 33 studies, insufficient reporting on designs. | Suicide specific treatment: evidence indicates that suicide specific treatments were effective in reducing both PTSD and suicide-related conditions. Brief cognitive behavioural therapy: reductions in both PTSD and suicide attempts compared to TAU, and at follow-up (one study. Postadmission cognitive therapy for the prevention of suicide (PACT) (2 studies). Reductions in both PTSD and suicide outcomes. DBT (4 studies), reductions in suicide-related symptoms. Cognitive processing therapy (11 studies) significantly reduced suicide-related outcomes. | Heterogeneity discussed in discussion over study samples, designs, and outcome measures. | Effective Public Health Practice Project quality assessment tool (Thomas et al., 2004): Overall, 10 studies were rated as strong, 17 studies rated as moderate, and six studies as weak.  Mixed settings, mostly military, but also inpatient and outpatient), mixed populations (e.g., history of self-harm, trauma, psychiatric diagnosis); complex variable interventions. Wide range of measurement for suicide outcomes, lack of underrepresented groups |

Abbreviations: CT= controlled trials; CCT = controlled cohort studies; CBT= cognitive behaviour therapy; CAT; Cognitive analytic therapy; CPDpd= Cognitive behaviour therapy adapted for people who have received a personality disorder diagnosis. CROC: Cochrane Risk of Bias tool; EUC= Enhanced usual care; MBT; Mentalisation based therapy; DBT= dialectical behaviour therapy; RCT=Randomised controlled trials; ITS= Interrupted times series designs; Obs.= observational study design; PP= Pre-post, QED= Quasi-experimental designs; TAU = treatment as usual; CROB: Cochrane Risk of Bias

**Included review references**

1. DeCou CR, Comtois KA, Landes SJ. Dialectical behavior therapy is effective for the treatment of suicidal behavior: A meta-analysis. *Behav Ther*. 2019;50(1):60-72.
2. Donker T, Calear A, Grant JB, Van Spijker B, Fenton K, Hehir KK, et al. Suicide prevention in schizophrenia spectrum disorders and psychosis: a systematic review. *BMC Psychol*. 2013;1:1-10.
3. Fox KR, Huang X, Guzmán EM, Funsch KM, Cha CB, Ribeiro JD, et al. Interventions for suicide and self-injury: A meta-analysis of randomized controlled trials across nearly 50 years of research. *Psychol Bull*. 2020;146(12):111.
4. Hawton K, Witt KG, Salisbury TLT, Arensman E, Gunnell D, Hazell P, et al. Psychosocial interventions following self-harm in adults: a systematic review and meta-analysis. *Lancet Psychiatry.* 2016;3(8):740-50.
5. Hou X, Wang J, Guo J, Zhang X, Liu J, Qi L, et al. Methods and efficacy of social support interventions in preventing suicide: a systematic review and meta-analysis. *BMJ Ment Health*. 2022;25(1):29-35.
6. Luxton DD, June JD, Comtois KA. Can postdischarge follow-up contacts prevent suicide and suicidal behavior? Crisis. 2013; Available from: <https://econtent.hogrefe.com/doi/10.1027/0227-5910/a000158>
7. Mann JJ, Michel CA, Auerbach RP. Improving suicide prevention through evidence-based strategies: a systematic review. Am J Psychiatry. 2021;178(7):611-24.
8. Nawaz RF, Reen G, Bloodworth N, Maughan D, Vincent C. Interventions to reduce self-harm on in-patient wards: systematic review. *BJPsych Open*. 2021;7(3):e80.
9. Rozek DC, Baker SN, Rugo KF, Steigerwald VL, Sippel LM, Holliday R, et al. Addressing co-occurring suicidal thoughts and behaviors and posttraumatic stress disorder in evidence-based psychotherapies for adults: A systematic review. *J Trauma Stress*. 2022;35(2):729-45.
10. Sobanski T, Josfeld S, Peikert G, Wagner G. Psychotherapeutic interventions for the prevention of suicide re-attempts: a systematic review. *Psychol Med*. 2021;51(15):2525-40.
11. Wand AP, Browne R, Jessop T, Peisah C. A systematic review of evidence-based aftercare for older adults following self-harm. *Aust N Z J Psychiatry*. 2022;56(11):1398-420.
12. Witt KG, Hetrick SE, Rajaram G, Hazell P, Salisbury TLT, Townsend E, et al. Psychosocial interventions for self-harm in adults—Witt, KG - 2021 | Cochrane Library. 2021. Available from: <https://www.cochranelibrary.com/cdsr/doi/10.1002/14651858.CD013668.pub2/full>
13. Yiu HW, Rowe S, Wood L. A systematic review and meta-analysis of psychosocial interventions aiming to reduce risks of suicide and self-harm in psychiatric inpatients. *Psychiatry Res*. 2021;305:114175.

| Table 4. Excluded reviews | |
| --- | --- |
| Reason for exclusion | Reference |
| Incorrect population and/or setting/intervention | 1. Arshad, U., Gauntlett, J., Husain, N., Chaudhry, N., & Taylor, P. J. (2020). A systematic review of the evidence supporting mobile‐and internet‐based psychological interventions for self‐harm. Suicide and Life‐Threatening Behavior, 50(1), 151-179. 2. Azizi, H., Esmaeili, E. D., Khodamoradi, F., & Sarbazi, E. (2022). Effective suicide prevention strategies in primary healthcare settings: a systematic review. Middle East Current Psychiatry, 29(1), 101. 3. Azizi, H., Fakhari, A., Farahbakhsh, M., Davtalab Esmaeili, E., Chattu, V. K., Ali Asghari, N., ... & Mansournia, M. A. (2023). Prevention of re-attempt suicide through brief contact interventions: a systematic review, meta-analysis, and meta-regression of randomized controlled trials. Journal of Prevention, 1-18. 4. Balcombe, L., & De Leo, D. (2022). The potential impact of adjunct digital tools and technology to help distressed and suicidal men: an integrative review. Frontiers in psychology, 12, 796371. 5. Baptista, M. N., Cunha, F. A., Batista, H. H. V., & Cremasco, G. D. S. (2022). Suicide prevention programs: An integrative literature review. Psicologia: Teoria e Prática, 24(2), 1-23. 6. Chalker, S. A., Martinez Ceren, C. S., Ehret, B. C., & Depp, C. A. (2022). Suicide-focused group therapy: A scoping review. Crisis: The Journal of Crisis Intervention and Suicide Prevention. 7. Chartier, G. B., Lam, F., Bergmans, Y., Lofchy, J., Bolton, J. M., Klonsky, E. D., ... & Kealy, D. (2023). “Psychotherapy in the Pressure Cooker”: A Systematic Review of Single Session Psychosocial Interventions in Emergency Departments for Suicide-related Thoughts or Behaviors. Journal of Psychiatric Practice, 29(4), 291-307. 8. Davidson, K. M., & Tran, C. F. (2014). Impact of treatment intensity on suicidal behavior and depression in borderline personality disorder: a critical review. Journal of Personality Disorders, 28(2), 181-197. 9. Dobias, M. L., Chen, S., Fox, K., & Schleider, J. L. (2020). Brief interventions for self-injurious thoughts and behaviors in young people: A systematic review. 10. Doupnik, S. K., Rudd, B., Schmutte, T., Worsley, D., Bowden, C. F., McCarthy, E., ... & Marcus, S. C. (2020). Association of suicide prevention interventions with subsequent suicide attempts, linkage to follow-up care, and depression symptoms for acute care settings: a systematic review and meta-analysis. JAMA psychiatry, 77(10), 1021-1030. 11. Frey, L. M., Hunt, Q. A., Russon, J. M., & Diamond, G. (2022). Review of family‐based treatments from 2010 to 2019 for suicidal ideation and behavior. Journal of marital and family therapy, 48(1), 154-177. 12. Hofstra, E., Van Nieuwenhuizen, C., Bakker, M., Özgül, D., Elfeddali, I., de Jong, S. J., & van der Feltz-Cornelis, C. M. (2020). Effectiveness of suicide prevention interventions: a systematic review and meta-analysis. General hospital psychiatry, 63, 127-140. 13. Lengvenyte, A., Olie, E., Strumila, R., Navickas, A., Gonzalez Pinto, A., & Courtet, P. (2021). Immediate and short-term efficacy of suicide-targeted interventions in suicidal individuals: a systematic review. The World Journal of Biological Psychiatry, 22(9), 670-685. 14. McCabe, R., Garside, R., Backhouse, A., & Xanthopoulou, P. (2018). Effectiveness of brief psychological interventions for suicidal presentations: a systematic review. BMC psychiatry, 18(1), 1-13. 15. Meerwijk, E. L., Parekh, A., Oquendo, M. A., Allen, I. E., Franck, L. S., & Lee, K. A. (2016). Direct versus indirect psychosocial and behavioural interventions to prevent suicide and suicide attempts: a systematic review and meta-analysis. The Lancet Psychiatry, 3(6), 544-554. 16. Milner, A., Witt, K., Pirkis, J., Hetrick, S., Robinson, J., Currier, D., ... & Carter, G. L. (2017). The effectiveness of suicide prevention delivered by GPs: a systematic review and meta-analysis. Journal of Affective Disorders, 210, 294-302 17. Noh, D., Park, Y. S., & Oh, E. G. (2016). Effectiveness of telephone-delivered interventions following suicide attempts: a systematic review. Archives of psychiatric nursing, 30(1), 114-119. 18. Nuij, C., van Ballegooijen, W., De Beurs, D., Juniar, D., Erlangsen, A., Portzky, G., ... & Riper, H. (2021). Safety planning-type interventions for suicide prevention: meta-analysis. The British Journal of Psychiatry, 219(2), 419-426. 19. Okolie, C., Dennis, M., Thomas, E. S., & John, A. (2017). A systematic review of interventions to prevent suicidal behaviors and reduce suicidal ideation in older people. International psychogeriatrics, 29(11), 1801-1824. 20. Pistone, I., Beckman, U., Eriksson, E., Lagerlöf, H., & Sager, M. (2019). The effects of educational interventions on suicide: A systematic review and meta-analysis. International journal of social psychiatry, 65(5), 399-412. 21. Tofthagen, R., Gabrielsson, S., Fagerström, L., Haugerud, L. M., & Lindgren, B. M. (2022). Men who self‐harm—A scoping review of a complex phenomenon. Journal of Advanced Nursing, 78(5), 1187-1211. 22. Yonemoto, N., Kawashima, Y., Endo, K., & Yamada, M. (2019). Gatekeeper training for suicidal behaviors: A systematic review. Journal of affective disorders, 246, 506-514. 23. Zarska, A., Barnicot, K., Lavelle, M., Dorey, T., & McCabe, R. (2023). A systematic review of training interventions for emergency department providers and psychosocial interventions delivered by emergency department providers for patients who self-harm. Archives of suicide research, 27(3), 829-850. 24. Navin K, Kuppili PP, Menon V, Kattimani S. Suicide Prevention Strategies for General Hospital and Psychiatric Inpatients: A Narrative Review. Indian Journal of Psychological Medicine. 2019 Sep 1;41(5):403–12. 25. Ferguson M, Rhodes K, Loughhead M, McIntyre H, Procter N. The Effectiveness of the Safety Planning Intervention for Adults Experiencing Suicide-Related Distress: A Systematic Review. Archives of Suicide Research. 2022 Jul 3;26(3):1022–45. |
| Not a systematic review | 1. Bakalar, J. L., Carlin, E. A., Blevins, C. L., & Ghahramanlou-Holloway, M. (2016). Generalizability of evidence-based PTSD psychotherapies to suicidal individuals: A review of the Veterans Administration and Department of Defense clinical practice guidelines. Military Psychology, 28(5), 331-343. 2. Brown, G.K. and Green, K.L., 2014. A review of evidence-based follow-up care for suicide prevention: where do we go from here?. American journal of preventive medicine, 47(3), pp.S209-S215. 3. Evans, N., Edwards, D., & Chick, P. (2022). Managing suicidality in inpatient care: a rapid review. The Journal of Mental Health Training, Education and Practice, 17(6), 479-494. 4. Fakhari, A., Azizi, H., Farahbakhsh, M., & Esmaeili, E. D. (2022). Effective programs on suicide prevention: combination of review of systematic reviews with expert opinions. International journal of preventive medicine, 13. 5. Fazel, S., & Runeson, B. (2020). Suicide. N Engl J Med 2020; 382:266-274 6. Gore, K. L., Chen, C., Fu, N., Larkin, J., Motala, A., & Hempel, S. (2023). Interventions for people who have attempted suicide and their family members: a systematic review. Rand health quarterly, 10(4). 7. Júlia, M. (2015). Telephone Contact as a Method of Prevention of Recurrence After a Suicidal Attempt: a Review. European Psychiatry, 30, 952. 8. Linskens, E. J., Venables, N. C., Gustavson, A. M., Sayer, N. A., Murdoch, M., MacDonald, R., ... & Sultan, S. (2022). Population-and Community-Based Interventions to Prevent Suicide. Crisis. 9. Martínez-Alés, G., & Keyes, K. M. (2019). Fatal and non-fatal self-injury in the USA: Critical review of current trends and innovations in prevention. Current psychiatry reports, 21, 1-11. 10. Platt, S., & Niederkrotenthaler, T. (2020). Suicide prevention programs. Crisis. 11. Rudd, B. N., Davis, M., Doupnik, S., Ordorica, C., Marcus, S. C., & Beidas, R. S. (2022). Implementation strategies used and reported in brief suicide prevention intervention studies. JAMA psychiatry, 79(8), 829-831. 12. Spafford, S. G. (2023). The Effects of Suicide Prevention Gatekeeper Training on Behavioral Intention, and Intervention Behavior: A Systematic Review and Meta-Analysis (Doctoral dissertation, University of Oregon). 13. Vázquez, L. C., Meca, A. C., Ruíz, A. S., Jiménez-Villamizar, M. P., Sanabria-Mazo, J. P., Canedo, C. M., & Vidal, D. P. (2022). Efficacy of Synchronous Remote-Based Interventions for Suicide Prevention among Adolescent and Adult Patients: A Systematic Review and Meta-Analysis. European Psychiatry, 65(S1), S295-S296. 14. Wallace, M., Miller, V. J., Fields, N. L., Xu, L., & Mercado-Sierra, M. A. (2021). Empirically evaluated suicide prevention program approaches for older adults: a review of the literature from 2009-2021. Journal of gerontological social work, 64(5), 480-498. |
| No relevant data for interventions | 1. Acosta, J., Ramchand, R., Jaycox, L. H., Becker, A., & Eberhart, N. K. (2013). Interventions to Prevent Suicide: A Literature Review to Guide Evaluation of California's Mental Health Prevention and Early Intervention Initiative. Rand Health Quarterly, 2(4). 2. da Silva, C. R., Gomes, A. A. D., dos Santos-Doni, T. R., Antonelli, A. C., da Costa Vieira, R. F., & da Silva, A. R. S. (2023). Suicide in veterinary medicine: A literature review. Veterinary world, 16(6), 1266. 3. Fernandez-Rodrigues, V., Sanchez-Carro, Y., Lagunas, L. N., Rico-Uribe, L. A., Pemau, A., Diaz-Carracedo, P., ... & de la Torre-Luque, A. (2022). Risk factors for suicidal behaviour in late-life depression: a systematic review. World journal of psychiatry, 12(1), 187. 4. Ghoncheh, R., Koot, H. M., & Kerkhof, A. J. (2014). Suicide prevention e-learning modules designed for gatekeepers. Crisis. 5. Holm, A. L., Salemonsen, E., & Severinsson, E. (2021). Suicide prevention strategies for older persons—An integrative review of empirical and theoretical papers. Nursing open, 8(5), 2175-2193. 6. King, K., Krysinska, K., & Nicholas, A. (2022). A rapid review to determine the suicide risk of separated men and the effectiveness of targeted suicide prevention interventions. Advances in Mental Health, 20(3), 184-199. 7. Kleiman, E. M., Bentley, K. H., Glenn, C. R., Liu, R. T., & Rizvi, S. L. (2021). Building on the past 50 years, not starting over: A balanced interpretation of meta-analyses, reviews, and commentaries on treatments for suicide and self-injury. General hospital psychiatry, 74, 18-21. 8. Legazpi, P. C. C., Rodríguez-Muñoz, M. F., Olivares-Crespo, M. E., & Izquierdo-Méndez, N. (2022). Review of suicidal ideation during pregnancy: risk factors, prevalence, assessment instruments and consequences. Psicologia: Reflexão e Crítica, 35. 9. Pearson, M., Zwi, A. B., Rouse, A. K., Fernando, R., Buckley, N. A., & McDuie-Ra, D. (2014). Taking Stock–What Is Known About Suicide in Sri Lanka. Crisis. 10. Ramsey, C., Galway, K., & Davidson, G. (2022). Implementing changes after patient suicides in mental health services: A systematic review. Health & Social Care in the Community, 30(2), 415-431. 11. Sufrate‐Sorzano, T., Pérez, J., Juárez‐Vela, R., Garrote‐Cámara, M., de Viñaspre, R. R., Molina‐Luque, F., & Santolalla‐Arnedo, I. (2023). Umbrella review of nursing interventions NIC for the treatment and prevention of suicidal behavior. International journal of nursing knowledge, 34(3), 204-215. 12. Witt, K., de Moraes, D. P., Salisbury, T. T., Arensman, E., Gunnell, D., Hazell, P., ... & Hawton, K. (2018). Treatment as usual (TAU) as a control condition in trials of cognitive behavioural-based psychotherapy for self-harm: impact of content and quality on outcomes in a systematic review. Journal of Affective Disorders, 235, 434-447. |
| Suicide/self-harm not primary outcome measure or composite measures | 1. Ferguson, M. S., Reis, J. A., Rabbetts, L., Ashby, H. J., Bayes, M., McCracken, T., ... & Procter, N. G. (2017). The effectiveness of suicide prevention education programs for nurses. Crisis. 2. Hanlon, C. A., McIlroy, D., Poole, H., Chopra, J., & Saini, P. (2023). Evaluating the role and effectiveness of co‐produced community‐based mental health interventions that aim to reduce suicide among adults: A systematic review. Health Expectations, 26(1), 64-86. 3. Hill, K., Somerset, S., Armstrong, D., Schwarzer, R., & Chan, C. (2022). Saving lives: A systematic review on the efficacy of theory-informed suicide prevention programs. Community mental health journal, 58(3), 454-473. 4. Jeong, H., Yim, H. W., Lee, S. Y., Potenza, M. N., & Kim, N. J. (2023). Effectiveness of Psychotherapy on Prevention of Suicidal Re-Attempts in Psychiatric Emergencies: A Systematic Review and Network Meta-Analysis of Randomized Controlled Trials. Psychotherapy and Psychosomatics, 1-10. 5. Kim, J., Grace, Y. E. H., Macy, R. J., Rizo, C. F., & Wretman, C. J. (2022). Interventions to prevent suicidality among survivors of intimate partner violence: a systematic review. Partner abuse, 13(3), 345-365. 6. Kwon, C. Y., & Lee, B. (2023, March). The effectiveness and safety of acupuncture on suicidal behavior: a systematic review. In Healthcare (Vol. 11, No. 7, p. 955). MDPI. 7. Lai, M. H., Maniam, T., Chan, L. F., & Ravindran, A. V. (2014). Caught in the web: a review of web-based suicide prevention. Journal of medical Internet research, 16(1), e30. 8. Malakouti, S. K., Rasouli, N., Rezaeian, M., Nojomi, M., Ghanbari, B., & Mohammadi, A. S. (2020). Effectiveness of self-help mobile telephone applications (apps) for suicide prevention: A systematic review. Medical journal of the Islamic Republic of Iran, 34, 85. 9. Abu Sabra, M. A., Al Kalaldeh, M., Khalil, M., Abualruz, H., & Hamdan‐Mansour, A. M. (2023). The efficacy of using psychotherapy treatments for obsessive–compulsive disorder on minimizing suicidal thoughts and behaviours: A scoping review. Clinical Psychology & Psychotherapy. 10. Petrie, K., Crawford, J., Baker, S. T., Dean, K., Robinson, J., Veness, B. G., ... & Harvey, S. B. (2019). Interventions to reduce symptoms of common mental disorders and suicidal ideation in physicians: a systematic review and meta-analysis. The Lancet Psychiatry, 6(3), 225-234. 11. Qu, D., Wen, X., Liu, B., Zhang, X., He, Y., Chen, D., ... & Chen, R. (2023). Non-suicidal self-injury in Chinese population: a scoping review of prevalence, method, risk factors and preventive interventions. The Lancet Regional Health–Western Pacific. 12. Tighe, J., Nicholas, J., Shand, F., & Christensen, H. (2018). Efficacy of acceptance and commitment therapy in reducing suicidal ideation and deliberate self-harm: systematic review. JMIR mental health, 5(2), e10732. 13. Zeppegno, P., Gattoni, E., Mastrangelo, M., Gramaglia, C., & Sarchiapone, M. (2019). Psychosocial suicide prevention interventions in the elderly: A mini-review of the literature. Frontiers in psychology, 9, 2713. |
| Not accessible in English | 1. Leiman, M., & Javier Garay, C. (2017). Psychological Interventions for Patients with Suicidal Risk: A Systematic Review. ACTA PSIQUIATRICA Y PSICOLOGICA DE AMERICA LATINA, 63(4), 261-275. 2. Penadés, R., Forte, F., Mezquida, G., González-Rodríguez, A., García-Rizo, C., Catalán, R., & Bernardo, M. (2022). Effectiveness of Cognitive Behavioural Therapy for Suicide in Schizophrenia: A Systematic Review. Current Psychiatry Research and Reviews Formerly: Current Psychiatry Reviews, 18(1), 36-45. 3. Pirola, A., Preti, E., Madeddu, F., Micucci, D., & Calati, R. (2022). Internet-and Mobile-based interventions aimed at suicide risks: a review of the literature. Recenti progressi in medicina, 113(4), 256-262. |
| Insufficient data | 1. Assing Hvidt, E., Ploug, T., & Holm, S. (2016). The impact of telephone crisis services on suicidal users: A systematic review of the past 45 years. Mental Health Review Journal, 21(2), 141-160. 2. Bowersox, N. W., Jagusch, J., Garlick, J., Chen, J. I., & Pfeiffer, P. N. (2021). Peer‐based interventions targeting suicide prevention: A scoping review. American journal of community psychology, 68(1-2), 232-248. 3. Büscher, R., Beisemann, M., Doebler, P., Micklitz, H. M., Kerkhof, A., Cuijpers, P., ... & Sander, L. B. (2022). Digital cognitive–behavioural therapy to reduce suicidal ideation and behaviours: a systematic review and meta-analysis of individual participant data. BMJ Ment Health, 25(e1), e8-e17. 4. Cuijpers, P., de Beurs, D. P., van Spijker, B. A., Berking, M., Andersson, G., & Kerkhof, A. J. (2013). The effects of psychotherapy for adult depression on suicidality and hopelessness: a systematic review and meta-analysis. Journal of affective disorders, 144(3), 183-190. 5. Harmon, L. M., Cooper, R. L., Nugent, W. R., & Butcher, J. J. (2016). A review of the effectiveness of military suicide prevention programs in reducing rates of military suicides. Journal of Human Behavior in the Social Environment, 26(1), 15-24. 6. Jiwatram-Negron, T., Brooks, M. A., Ward, M., & Meinhart, M. (2023). Systematic review of interventions to address suicidal behavior among people with a history of intimate partner violence: Promises and gaps across the globe. Aggression and Violent Behavior, 101871. 7. Knipe, D., Padmanathan, P., Newton-Howes, G., Chan, L. F., & Kapur, N. (2022). Suicide and self-harm. The Lancet, 399(10338), 1903-1916. 8. Mohatt, N. V., Begay, R. L., Goss, C. W., Shore, J. H., Kaufman, C. E., & Hicken, B. L. (2023). A scoping review of veteran suicide prevention programs in Native American communities and in the general population. Psychological services, 20(S1), 19. 9. Procter, N., Othman, S., Jayasekara, R., Procter, A., McIntyre, H., & Ferguson, M. (2023). The impact of trauma‐informed suicide prevention approaches: A systematic review of evidence across the lifespan. International journal of mental health nursing, 32(1), 3-13. 10. Sullivan, S. R., Spears, A. P., Mitchell, E. L., Walsh, S., Love, C., & Goodman, M. (2021). Family treatments for individuals at risk for suicide: A PRISMA scoping review. Crisis: The Journal of Crisis Intervention and Suicide Prevention. 11. Wu, H., Lu, L., Qian, Y., Jin, X. H., Yu, H. R., Du, L., ... & Chen, H. L. (2022). The significance of cognitive-behavioral therapy on suicide: An umbrella review. Journal of affective disorders, 317, 142-148. 12. Zalsman, G., Hawton, K., Wasserman, D., van Heeringen, K., Arensman, E., Sarchiapone, M., Carli, V., Höschl, C., Barzilay, R., Balazs, J. and Purebl, G., 2016. Suicide prevention strategies revisited: 10-year systematic review. The Lancet Psychiatry, 3(7), pp.646-659. |
